# Cyclometalated Osmium Compounds and beyond: Synthesis, Properties, Applications

**DOI:** 10.3390/molecules26061563

**Published:** 2021-03-12

**Authors:** Ricardo Cerón-Camacho, Manuel A. Roque-Ramires, Alexander D. Ryabov, Ronan Le Lagadec

**Affiliations:** 1 CONACyT-Instituto Mexicano del Petróleo, Eje Central Lázaro Cárdenas 152, Col. San Bartolo Atepehuacán, México D.F. 07730, Mexico; rceron@imp.mx; 2 Instituto de Química, UNAM, Circuito Exterior s/n, Ciudad Universitaria, Coyoacán, Ciudad de México 04510, Mexico; manuel.roque@comunidad.unam.mx; 3 Department of Chemistry, Carnegie Mellon University, 4400 Fifth Avenue, Pittsburgh, PA 15213, USA; ryabov@andrew.cmu.edu

**Keywords:** osmium, cyclometalation, osmium cyclometalated complex, osmacycle, pincer complex, catalysis, photophysics, anticancer activity

## Abstract

The synthesis of cyclometalated osmium complexes is usually more complicated than of other transition metals such as Ni, Pd, Pt, Rh, where cyclometalation reactions readily occur via direct activation of C–H bonds. It differs also from their ruthenium analogs. Cyclometalation for osmium usually occurs under more severe conditions, in polar solvents, using specific precursors, stronger acids, or bases. Such requirements expand reaction mechanisms to electrophilic activation, transmetalation, and oxidative addition, often involving C–H bond activations. Osmacycles exhibit specific applications in homogeneous catalysis, photophysics, bioelectrocatalysis and are studied as anticancer agents. This review describes major synthetic pathways to osmacycles and related compounds and discusses their practical applications.

## 1. Introduction

Osmium is the heaviest and rarest stable element in the earth’s crust, with a concentration of approximately 0.05 part per billion. Perhaps the latter accounts for why the coordination and organometallic chemistry of osmium are less advanced than that of other transition metals. Common precursors, such as OsCl_3_ or M_2_[OsCl_6_], are significantly more expensive than their respective ruthenium precursors. Osmium is often associated with highly toxic OsO_4,_ widely used as an oxidizing agent in organic chemistry and as a staining agent in transmission and scanning electron microscopies [1,2,3]. Fortunately, osmium chemistry is not limited to its oxides, and numerous coordination, organometallic, and cluster compounds have been prepared, though their chemistry is not as deeply explored as that of ruthenium [4]. The osmium chemistry was not extensively reviewed in the literature. The first review was written by Griffith in 1987 [5]. Some osmium compounds were also compiled in *Gmelin Handbuch der Anorganischen Chemie* in the 1980s [6]. In the 2011 edition of the *Encyclopedia of Inorganic Chemistry,* two articles were devoted to coordination and organometallic chemistry of osmium [7,8]. To our knowledge, the chemistry of cyclometalated osmium species was not specifically reviewed, in contrast with the extended coverage of the chemistry of other metalacycles in the last several decades [9,10,11,12,13,14,15,16,17,18,19,20,21,22,23].

**Figure 1:**
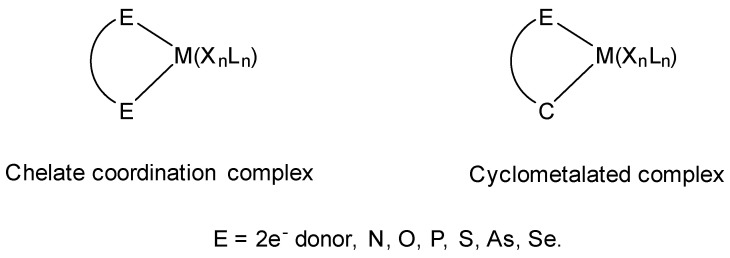
Chelate coordination and cyclometalated complexes.

This review attempts to fill the existing gap in covering the cyclometalation chemistry of osmium compounds as they are presented in Figure 1 with M = Os. The metal is usually σ-bonded to a carbon atom and coordinated to E, where E is generally N, P, O, S, As, Se. Undoubtedly, the impact of Michel Pfeffer’s work on the development of cyclometalation reactions was fundamental, and many of his publications, first on palladium then on ruthenium metalacycles, set the path for countless publications [24,25,26,27].

## 2. Historical Background

**Figure 2:**
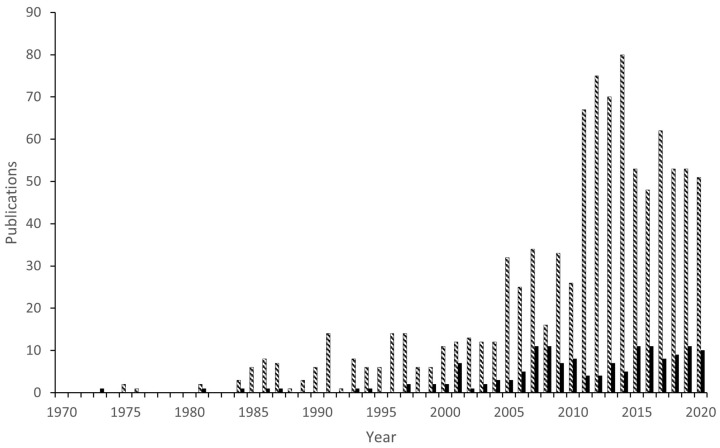
Year progression of publications mentioning osmacycles (black) and ruthenacycles (grey) (retrieved December 2020, SciFinder with keywords: osmacycle, osmium AND cyclometalated, osmium AND cyclometallated, cyclometalated AND osmium, metalacycle AND osmium or ruthenacycle, ruthenium AND cyclometalated, cyclometallated AND ruthenium, cyclometalated AND ruthenium, metalacycle AND ruthenium).

Ruthenium and osmium belong to group 8 metals, and they frequently form similar complexes. The oxidation states vary from 0 to VIII, though more common are oxidation states between II to IV [4]. Osmium forms stronger metal-metal and metal-carbon bonds, and hence osmium complexes are generally less active catalysts than analogous ruthenium species. Many osmium complexes are made via different or more difficult routes than their ruthenium analogs. Publications on osmacycles started to grow after 2000, prior to which they were nearly nonexistent. Their number is considerably lower than the number of reports on ruthenacycles (Figure 2).

The first osmacycle prepared by Bruce in 1973 was made from [Os_3_(CO)_12_] and benzo[*h*]quinoline. A mixture of mononuclear isomers **1** was obtained in low yields after 230 h at reflux in light petroleum [28]. In 1981, Jameson described cyclometalated formazan derivatives of osmium **2** [29]. Desrosiers prepared a hydride osmium–phosphine complex **3** by intramolecular C−H activation in 1986 [30]. Cyclometalated piano-stool derivative of benzoic acid **4** was reported by Kisenyl in 1987 [31]. Later, mononuclear and dinuclear pincer osmium complexes **5**, **6**, dinuclear Ru-Os cyclometalated derivative **7** [32,33], cationic mononuclear osma(II)cycle with PPh_3_ **8** [34], and osmacycle **9** from polyquinoline [35] were obtained. Structures of compound **5**–**11** are shown in Table 1.

## 3. Main Precursors

### 3.1. [OsX_2_(PR_3_)_3_]

Complexes [OsX_2_(PR_3_)_3_], a family of 16 electron osmium(II) precursors, are widely used for the synthesis of osmacycles. Among them, [OsBr_2_(PPh_3_)_3_] is particularly valuable.

#### 3.1.1. *mono*-Osmacycles

**Figure 3:**
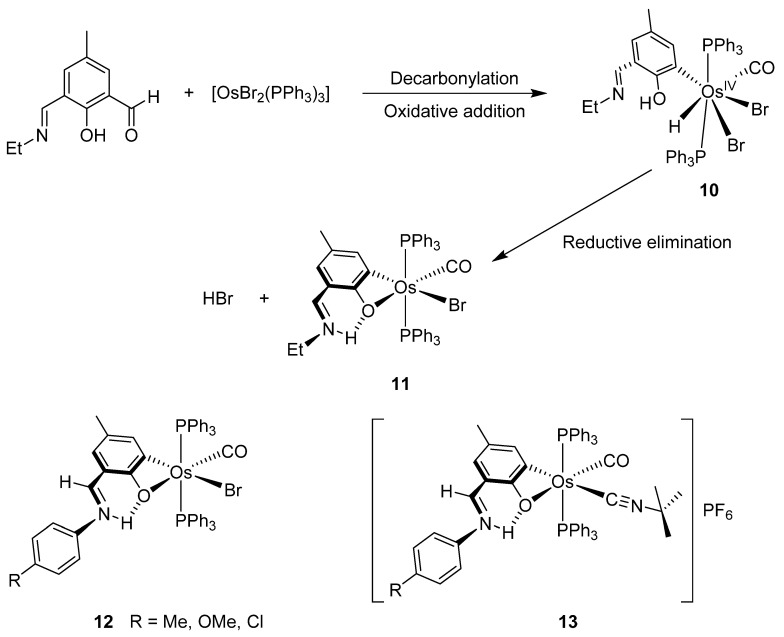
Synthesis of C~O cyclometalated complexes.

Chakravorty synthesized phenolate osmium complex **11** from 2,6-diformyl-4-methylphenol and [OsBr_2_(PPh_3_)_3_] [36]. The authors proposed the oxidative addition of the aldehyde, followed by decarbonylation, to form an unstable Os^IV^ intermediate. Subsequent reductive elimination of HX yields the cyclometalated complex [36]. Related [OsBr(C~O)(PPh_3_)_2_(CO)] complexes **12** were similarly prepared, and the substitution of bromide generated cationic species **13** (Figure 3) [37].

**Figure 4:**
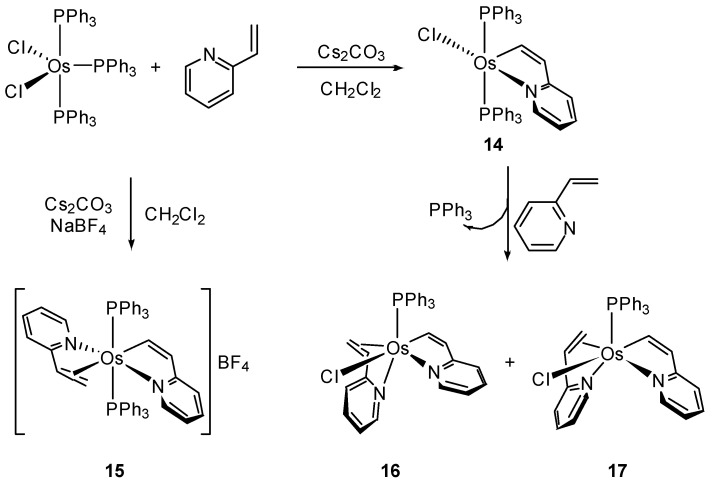
Cyclometalated complexes derived from 2-vinylpyridine.

The mechanism of cyclometalation of 2-vinylpyridine by [MCl_2_(PPh_3_)_3_] (M = Ru, Os) was explored theoretically for the ruthenium case [38]. The lower reactivity of osmium allowed to isolate several intermediates proposed. The reaction of [OsCl_2_(PPh_3_)_3_] with an excess of 2-vinylpyridine in the presence of Cs_2_CO_3_ and NaBF_4_ results in cyclometalation of the ligand with liberation of PPh_3_ to produce **14**. The cationic complex **15** is formed via substitution of the chloride by a second equivalent of 2-vinylpyridine in presence of NaBF_4_. In the absence of NaBF_4_, the neutral cyclometalated complex **14** generates isomers **16** and **17** (Figure 4) [38].

#### 3.1.2. Pincer and *bis*-Cyclometalated Complexes

**Figure 5:**
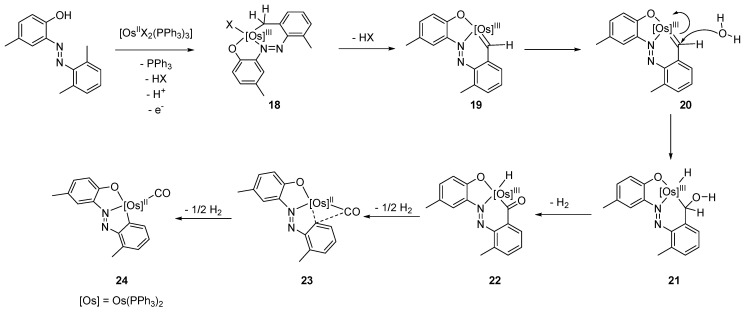
Synthesis of C~N~O pincer complexes.

[OsBr_2_(PPh_3_)_3_] reacts with 2-(2′,6′-dimethylphenylazo)-4-methylphenol in refluxing 2-methoxiethanol in the presence of NEt_3_ to produce osmium(III) complex **24**. The authors consider a coordination of the ligand to osmium(II) in a tridentate C~N~O fashion, followed by the oxidation to osmium(III) in **18** (Figure 5). Thereafter, the loss of HX affords a carbene species **19**. In the presence of water, the carbene intermediate could transform into the OsH-C=O fragment (**22**) after elimination of H_2_ in intermediate **21**. A posterior reductive elimination of a half equivalent of hydrogen from **22**, followed by the migration of a CO fragment (**23**), would produce the final cyclometalated complex [Os(C~N~O)(CO)] (**24)** [39].

**Figure 6:**
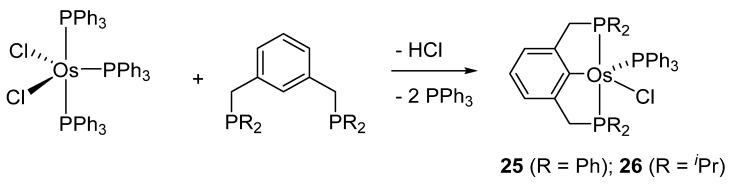
Synthesis of P~C~P pincer complex from 1,3-(R_2_PCH_2_)_2_C_6_H_4_.

Wen and co-workers prepared cyclometalated [Os(P~C~P)Cl(PPh_3_)] complexes **25** and **26** (Figure 6) [40]. They react with H_2_ to afford dihydrido species. The corresponding carbonyl derivatives can also be prepared in the presence of CO [41].

**Figure 7:**
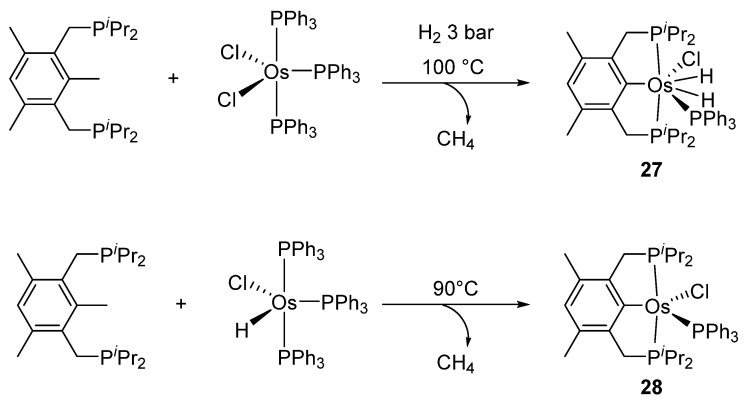
Synthesis of P~C~P pincer complexes by cleavage of a C-C bond.

The diphosphine {1,3,5-(CH_3_)_3_-2,6-(*^i^*Pr_2_-PCH_2_)_2_C_6_H} was also reacted with [OsCl_2_(PPh_3_)_3_]. The metal promotes the cleavage of one C−CH_3_ bond with liberation of methane (Figure 7). In the presence of H_2_, dihydride heptacoordinated complex **27** is formed, while [OsH(Cl)(PPh_3_)_3_] generates a pentacoordinated complex **28** [41].

**Figure 8:**
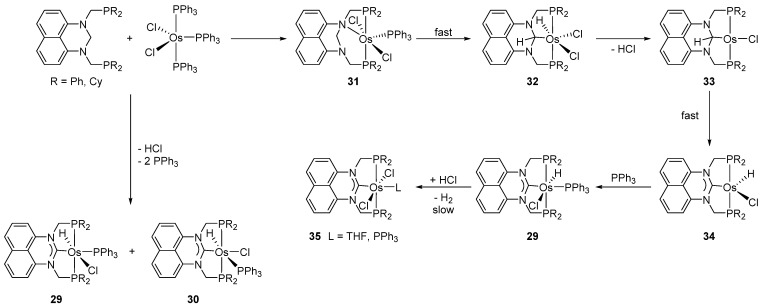
Synthetic route for a series of P~C~P pincer complexes by a double C−H activation.

The *N,N*’-bis(phosphinomethyl)dihydropermidine-type ligands H_2_C(NCH_2_PR_2_)_2_C_10_H_6_ (R = Cy, Ph) react with [OsCl_2_(PPh_3_)_3_] yielding the P~C~P pincer complexes **29** and **30**. The latter may undergo the second C−H activation to form the corresponding carbenes. Figure 8 shows the proposed mechanism for the double C−H activation [42].

**Figure 9:**
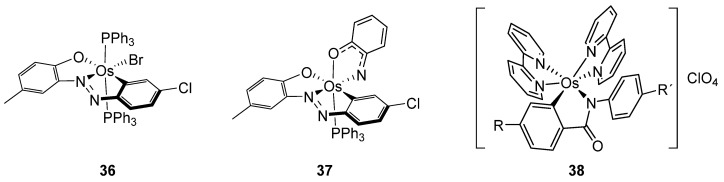
Cyclometalated osmium complexes obtained by C–H bond activation of phenols and hydroxamic acids.

Other examples involving C−H activation are shown in Figure 9. Majumder et al. synthesized osmacycles **36** and **37** from 2-(arylazo)phenols and [OsBr_2_(PPh_3_)_3_] [43], and Das found an unusual cyclometalation of *N*-arylbenzohydroxamic acids leading to **38** [44].

**Figure 10:**
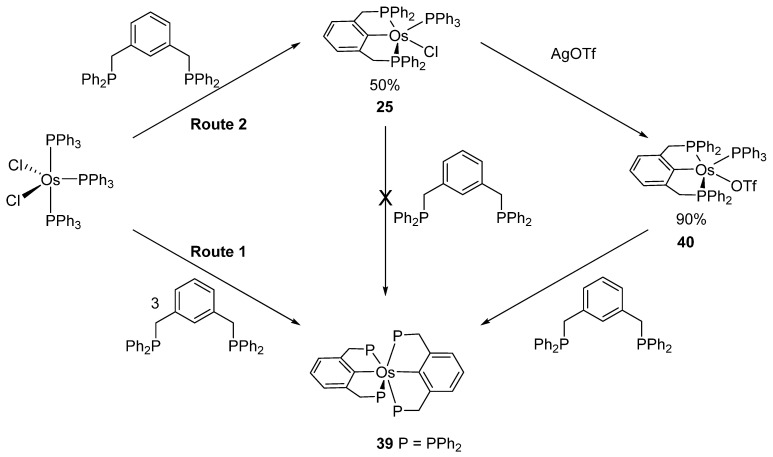
Synthesis of *bis*-(P~C~P) pincer complexes from 1,3-bis(diphenyl-phosphinomethyl)benzene.

*Bis*-pincer complex **39** was obtained from [OsCl_2_(PPh_3_)_3_] and diphosphine 2,6-(CH_2_PPh_2_)_2_C_6_H_3_ at a ligand-to-complex ratio of 3:1 (Route 1 in Figure 10). On the other hand, the ratio of 1:1 provides *mono*-pincer complex **25**. An exchange of chloride by triflate affords **40** and a subsequent reaction with the second equivalent of the ligand leads to *bis*-pincer complex **39** (Route 2 in Figure 10) [45].

#### 3.1.3. Others Osmacycles

**Figure 11:**
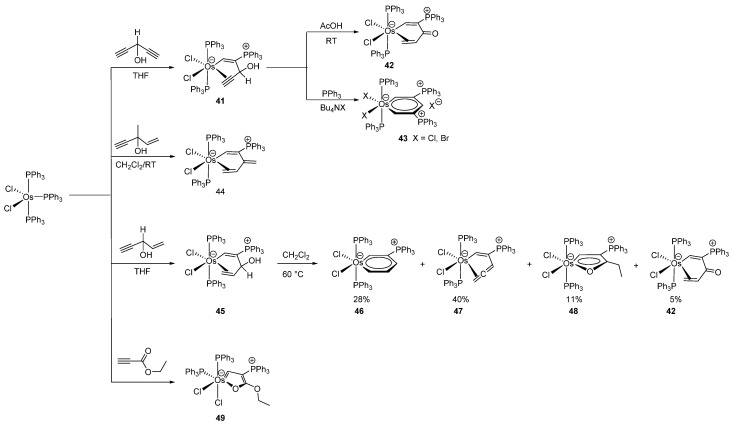
Unusual osmacycles prepared by activation of alkynes.

Gong studied the interaction between HC≡CCH(OH)CH=CH_2_ and [OsCl_2_(PPh_3_)_3_] in THF leading to η^2^-allyl alcohol osmacycle complexes, which could further be converted to osmabenzene, cyclic osmium η^2^-allene, osmafuran and α,β-unsaturated ketone complexes in a one-pot reaction [46] (Figure 11). It is worth mentioning that the “donor center” in such osmacycles is a C=C double bond, narrowly fitting into the definition of a metalacycle. Nevertheless, the authors classify the complexes as osmacycles. The cyclometalation takes place through a nucleophilic attack of PPh_3_ at the coordinated alkyne [47]. This method results in efficient preparations of conjugated osmacycles in high yields and allows the synthesis of complexes containing a phosphonium moiety incorporated in the metalacycle. Osmabenzenes were prepared in a similar way [47]. The η^2^-alkyne-coordinated alcohol complex **41** can be transformed under acidic conditions to a η^2^-coordinated α,β-unsaturated ketone **42**. Additionally, **41** reacted with triphenylphosphine to give **43** in presence of Bu_4_NX (X = Cl, Br) with elimination of OH^-^. The efficient cyclometalation to give **45** with η^2^-coordinated allyl alcohol was also performed. Upon thermal treatment, **45** converts to a mixture of four conjugated osmacycles (**42**, **46**–**48**) [48]. Osmafuran **49** was prepared similarly [48] (Figure 11).

### 3.2. [OsX_2_(CO)(PR_3_)_3_]

**Figure 12:**
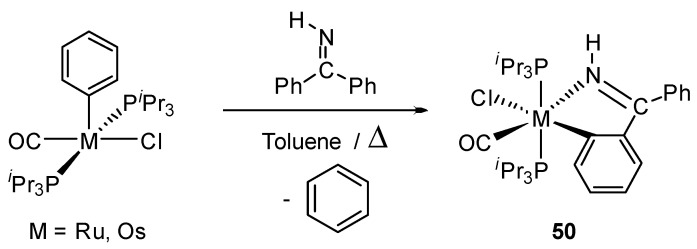
Synthesis of cyclometalated complex by oxidative addition of the phenyl group of an imine and subsequent reductive elimination.

Related precursors [OsX_2_(CO)(PR_3_)_2_] were also explored. The oxidative addition of the ortho C−H bond of the phenyl group of imine Ph_2_C=NH to [Os(Cl)(Ph)(P*^i^*Pr_3_)_2_(CO)] followed by the reductive elimination of benzene and the coordination of the iminic nitrogen generates osmacycle **50** (Figure 12). The process could be viewed as an unusual σ-bond metathesis [49].

**Figure 13:**
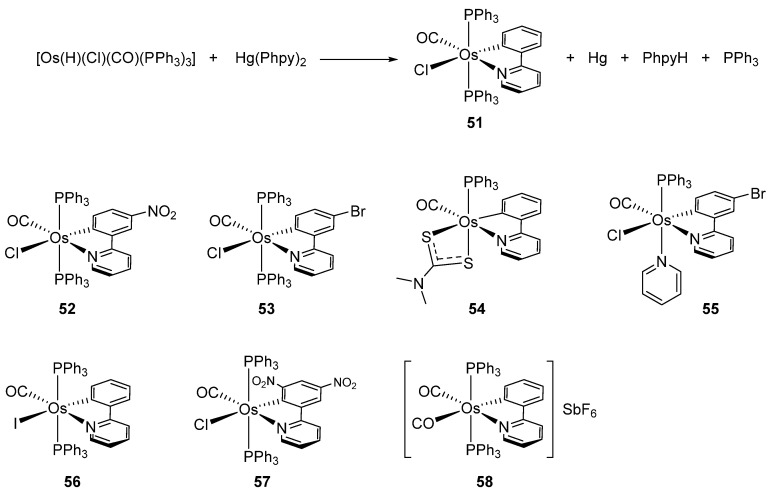
Osmium(II) complexes with cyclometalated phenylpyridines.

Clark et al. studied by X-ray crystallography four cyclometalated osmium complexes (**51**–**55**) obtained via the transmetalation reaction between hydride precursors [OsH(Cl)(CO)(PPh_3_)_3_] and organomercurial derivatives of 2-phenylpyridine. A series of related complexes (**56**–**58**) in which the phenylpyridine ligand was modified by electrophilic substitutions was also reported (Figure 13) [50].

**Figure 14:**
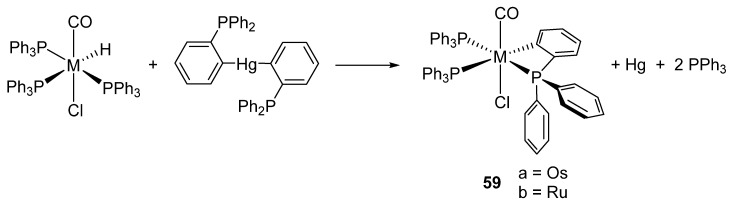
Use of organomercurial derivative to obtain cyclometalated complexes.

Bennett et al. also showed that the reaction between [Hg(*o*-C_6_H_4_PPh_2_)_2_] and [MH(Cl)(CO)(PPh_3_)_3_] (M = Ru, Os) form the corresponding four-membered metalacycles **59**, elemental mercury and free triphenylphosphine (Figure 14) [51]. It was suggested that the reaction occurs through the oxidative addition of Hg−Ar to a coordinatively unsaturated intermediate generated by the dissociation of PPh_3_ and the posterior reductive elimination of ArH and Hg [52].

### 3.3. Osmium Hydride Complexes

Esteruelas and coworkers have extensively been working on C−H bond activations mediated by transition metals. Their results represent an important step in the development of new synthetic routes through C−H bond activations by osmium. Notably, osmium hydrides complexes favor high oxidation states (4 and 6) and display a wider range of stoichiometries and structures than ruthenium derivatives. Osmium hydride complexes can also exhibit lability of the hydride ligands under thermal conditions [53]. Particularly, the hexahydride osmium(VI) precursor, [OsH_6_(P*^i^*Pr_3_)_2_], was widely used to prepare a variety of osmacycles. For example, this hexahydride osmium complex is capable of generating a tetrahydride [Os^IV^H_4_(P*^i^*Pr_3_)_2_] species and one hydrogen molecule by thermal activation [53]. The tetrahydride complex can then coordinate the oxygen atom of the carbonyl group of an aromatic ketone and subsequently activates C−H or C−F bonds for the corresponding cyclometalation step [54].

#### 3.3.1. [OsH_6_(PR_3_)_2_]

Studies by NMR revealed that [OsH_6_(P*^i^*Pr_3_)_2_] reacts with cycloalkyl or phenyl methyl ketone to form cyclometalated compounds **60**, **61** and **62** (Table 2) [49,50,54,55]. The reactivity of **61** and related complexes will be discussed in Section 4.2. The reaction of [OsH_6_(P*^i^*Pr_3_)_2_] with 2-vinylpiridine affords **63**, and a secondary C−H bond activation generates complex **64** [56]. The C(sp^3^)−H activation occurs when [OsH_6_(P*^i^*Pr_3_)_2_] reacts with 8-methylquinoline. Activation of the methyl group and elimination of two H_2_ molecules gives **65**. Experiments with methyl deuterated 8-methylquinoline and DFT calculations confirmed the mechanism [57].

The same hexahydride osmium precursor in the presence of 0.5 eq of 2,6-bis{1-[(4-methylphenyl)imino]ethyl}pyridine is involved in a double C−H activation of the pyridine ring producing a dimetalatricyclic system **66** (Table 2). The product was characterized by an X-ray study and the presence of three fused rings was confirmed [58]. Other bimetallic osmacycles were prepared from [OsH_6_(P*^i^*Pr_3_)_2_] by activation of the *ortho* C−H bond of *N,N’*-di- and tetraphenyl bipyridines. Starting from 2-phenylpyridine, compound **67** was first obtained (Table 2). Using 2,2′-diphenyl-4,4′-bipyridine in 1:3 and 3:1 ratios with respect to osmium allowed to isolate mononuclear **68** and dinuclear **69** osmacycles, respectively (Table 2). The corresponding phenyl and anthracenyl derivatives of bipyridine also form dinuclear osmacycles (**70**, **71**) [59]. Complex [OsH_6_(P*^i^*Pr_3_)_2_] and 2-azetidinones substituted at 4-position by an *N*-heterocycle and in 3-position by a phenoxy group afford corresponding cyclometalated complexes (**72**, **73**). This reaction is associated with the metal-mediated degradation of the ligand which involves two C−H bond activations and a parallel C−N and C−C rupture within the four-membered rings [60,61] (Table 2). Double cyclometalation takes place when [OsH_6_(P*^i^*Pr_3_)_2_] reacts with 2,6-diphenylpyridine to give tridentate C~N~C pincer compound **74**. In the presence of HBF_4_ the latter can suffer the addition of one proton at the metalated carbon to form the cationic *mono*-cyclometalated complex, which eliminates 2,6-diphenylpyridine in the presence of acetonitrile to generate the corresponding coordination derivative. When the ligand is 2-phenoxy-6-phenylpyridine, a double C−H activation also occurs. Five- and six-membered rings **75** are produced (Table 2) [62]. Other reactions with [OsH_6_(P*^i^*Pr_3_)_2_] involve the cyclometalation of azole ligands by selective C−H bond activation which can discern the coordination of N over “N−H side” in the ligand (**76**, **77**). Such behavior is also observed with 2-phenylthiazole and 2-phenylbenzothiazole, where the cyclometalation occurs at the “C−N side” [63]. The reaction of the hexahydride precursor with benzonitrile in toluene promotes the C−H activation and the reduction of the triple C≡N bond forming the corresponding imine. The resulting complex **78** is produced in a 75% yield. The substitution of P*^i^*Pr_3_ by PPh_3_ in such complexes was also studied [64]. Complex [OsH_6_(P*^i^*Pr_3_)_2_] can also activate directly the 8-position in 2-, 3-, 6- and 7-methylquinolines to obtain corresponding [OsH_3_{κ^2^-C^8^,N-(quinilinyl)}(P*^i^*Pr_3_)_2_] and [OsH_3_{κ^2^-C^8^,*N*-(quinilinyl-*n*-Me)}(P*^i^*Pr_3_)_2_] (**79**, *n* = 2, 3, 6, 7) complexes. It is interesting to note that a four-membered cyclometalated fragment is formed in all cases (Table 2) [65]. Complex [OsH_6_(P*^i^*Pr_3_)_2_] reacts with 2-vinylpyridine to form an osmacycle **80** where C(sp^2^)−H activation of the vinyl is observed (Table 2) [56].

**Figure 15:**
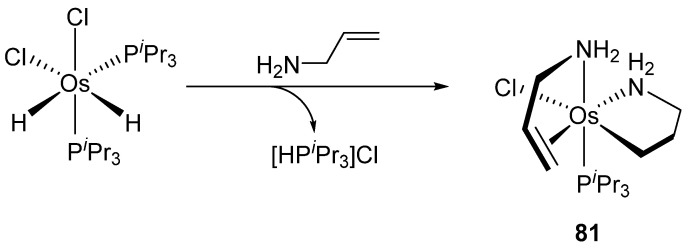
Reaction between [OsH_2_Cl_2_(P*^i^*Pr_3_)_2_] and allylamine.

A curious reductive cyclometalation occurs when the dihydride 16e Os^IV^ complex [OsH_2_Cl_2_(P*^i^*Pr_3_)_2_] reacts with an excess of allylamine. Cyclometalated Os^II^ species **81** is formed alongside with the [HP*^i^*Pr_3_]Cl salt (Figure 15). The final product is coordinated by two molecules of allyl amine. One of them is cyclometalated through the insertion of the alkene fragment into the Os−H bond. The mechanism proposed by the authors suggests a C−H activation to justify the [HP*^i^*Pr_3_]Cl formation. This cyclometalated complex **81** is the starting material to form a variety of other complexes by either ligand metathesis or CO and allene insertions [66].

**Figure 16:**
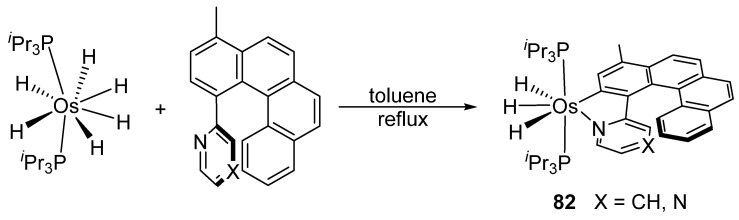
Cyclometalation via by C–H activation assisted by reductive elimination of hydride ligands.

The cyclometalation of helicenes occurs similarly (Figure 16). [OsH_2_Cl_2_(P*^i^*Pr_3_)_2_] reacts with the ligand and the cyclometalation to form **82** is accompanied by the reduction of the metal center to Os^IV^ in refluxing toluene [67].

#### 3.3.2. *bis*- and *tris*-Osmacycles

**Figure 17:**
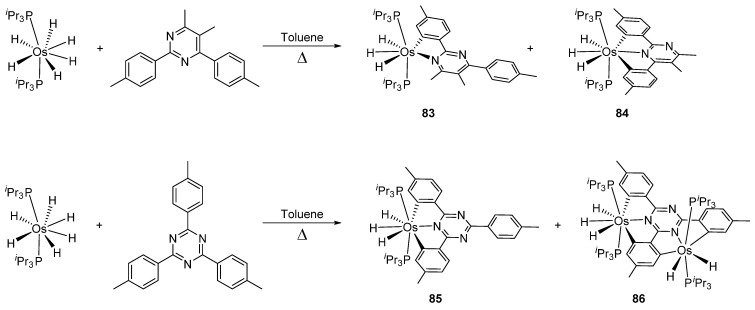
Synthesis of *bis*-cyclometalated complexes from [OsH_6_(P*^i^*Pr_3_)_2_].

Several osmacycles that contain two osmium–carbon σ-bonds (*bis*-cyclometalated), as well as some rare examples with three osmium–carbon σ-bonds (*tris*-cyclometalated) were prepared from [OsH_6_(P*^i^*Pr_3_)_2_]. The first examples were classified by Esteruelas et al. as “multiple C−H bond activations”. Complex [OsH_6_(P*^i^*Pr_3_)_2_] reacts with phenyl-substituted pyrimidines or triazines to form the corresponding [Os(C~N~C)] pincer derivatives. The reaction with phenyl pyrimidines leads to *mono*-cyclometalated complexes along with the pincer species. The phenyl triazine ligand forms pincer complex **85** but can also generate a dinuclear *bis*-cyclometalated osmium complex **86** (Figure 17) [68].

**Figure 18:**
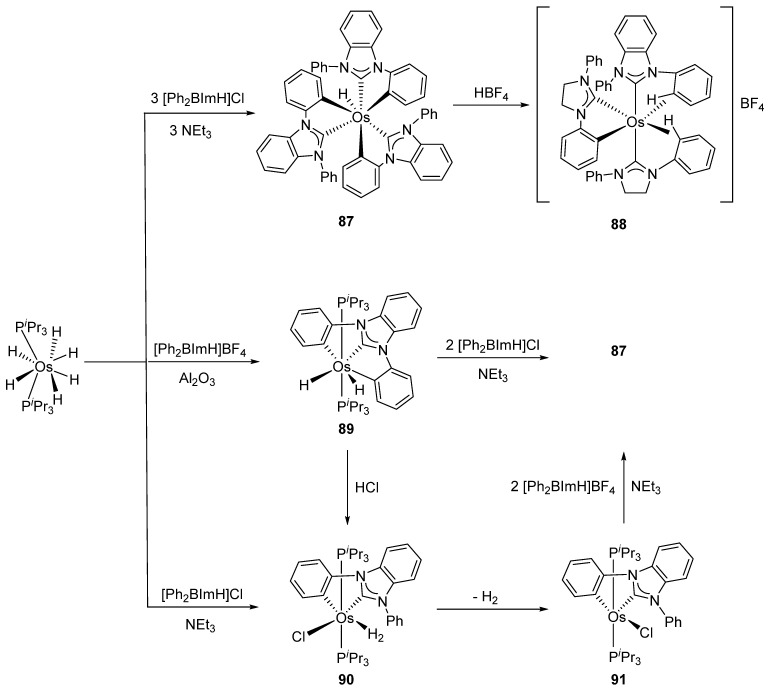
Synthetic routes to *tris*-cyclometalated complexes from *mono*-cyclometalated derivatives.

More recently, Esteruelas et al. described *tris*-cyclometalated osmium complex **87** produced from 3 eq of *N,N*’-diphenylbenzimidazolium chloride [Ph_2_BImH]Cl and [OsH_6_(P*^i^*Pr_3_)_2_] in the presence of a base. In acidic medium (HBF_4_), one cyclometalated phenyl group of **87** is protonated and a reductive elimination of hydride with another phenyl group forms a cationic octaedral complex **88** stabilized by two agostic interactions between osmium and C−H bonds (Figure 18) [69]. The replacement of chloride by BF_4_^-^ in the *N,N*’-diphenylbenzimidazolium salt promoted the tridentate coordination to form **89**. Monocyclometalated complex **90** was made using of one equivalent of [Ph_2_BImH]Cl. However, complex **87** could be obtained through the subsequent reaction with another equivalent of the ligand [69].

### 3.4. [Os(η^6^-arene)Cl_2_]_2_

The [Os(η^6^-arene)Cl_2_]_2_ dimers usually display similar reactivity as their widely used ruthenium analogues. The bridging chloride bonds are readily broken in polar solvents or in the presence of coordinating ligands. Monomers formed were used to prepare a variety of osmacycles [70].

#### 3.4.1. *mono*-Osmacycles

**Figure 19:**
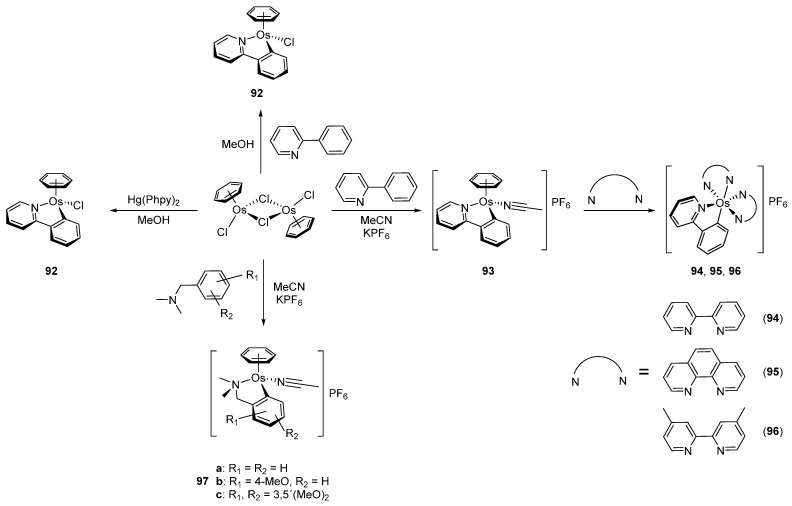
Cyclometalated osmium complexes obtained from [Os(η^6^-C_6_H_6_)(μ-Cl)Cl]_2_.

In 2003, piano-stool neutral **92** and cationic **93** osmacycles were prepared from the dimeric [Os(η^6^-C_6_H_6_)(μ-Cl)Cl]_2_ precursor (Figure 19) [71]. We are particularly proud that the first experiments were carried out in the lab of Michel Pfeffer in Strasbourg. Needless to say that background for this work was previously created by Michel through the thorough investigation of the chemistry of matching ruthenium complexes [72,73,74,75]. Coordinated benzene and acetonitrile in **93** are easily substituted by 2,2′-bipyridines or 1,10-phenantroline to form octahedral cationic complexes (**94**, **95**, **96**) [71]. Complex **92** can also be synthetized through a transmetalation reaction with organomercurial derivatives and the [Os(η^6^-C_6_H_6_)(μ-Cl)Cl]_2_ dimer [71]. Similarly, series of complexes bearing cyclometalated 2-phenylpyridine and *N,N*-dimethylbenzylamines with different electron-donating groups such as 4-OMe (**97b**) and 3,5-(OMe)_2_ (**97c**) were prepared with the aim to modulate the electron density at the osmium center [76].

**Figure 20:**
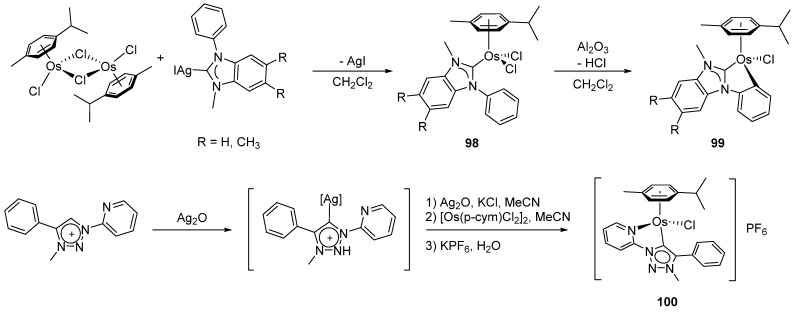
Synthesis of *mono*-cyclometalated complexes through transmetalation reactions with silver(I) derivatives.

Transmetalation between [Os(η^6^-C_6_H_6_)(μ-Cl)Cl]_2_ and silver(I) compounds is exemplified by the reaction of the 1-phenyl-3-methyl-1H-benzimidazolium [AgPhMeBIm]I complex. Intermediate **98** forms first followed by the metalation of the phenyl group in the presence of Al_2_O_3_. Piano-stool *mono*-cyclometalated complex **99** is the final product (Figure 20) [77]. It takes two days for triazolium silver salts to react with [Os(η^6^-C_6_H_6_)(μ-Cl)Cl]_2_ to give structurally similar complex **100** after treatment with KPF_6_ [78]. This methodology was also successfully employed to prepare cyclometalated complexes of iridium and ruthenium [78].

#### 3.4.2. *bis*- and *tris*-Osmacycles

**Figure 21:**
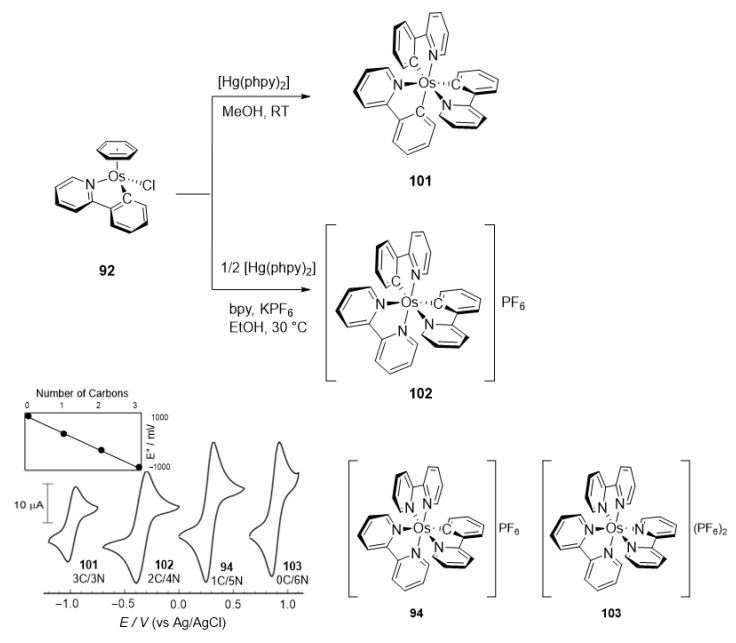
Synthesis of *bis*- and *tris*-cyclometalated complexes (top) and cyclic voltammograms showing the influence of the number of Os−C bonds on the Os^II^/Os^III^ reduction potentials). Bottom: cyclic voltammograms of **103**, **94**, **101** and **102** (1 mM) in MeCN: 25 °C, scan rate 0.1 V s^−1^, 0.1 M *^n^*Bu_4_NPF_6_, glassy carbon electrode. Inset shows a plot of the formal reduction potential vs. the number of C donors.

A few *bis*- and *tris*-cyclometalated osmium complexes have been described [79,80]. Such compounds differ from pincer complexes, as they contain two or three cyclometalated ligands, respectively. Complexes [Os(phpy)_2_(bpy)]PF_6_ (**102**) and [Os(phpy)_3_] (**101**) were prepared from organomercurial Hg(phpy)_2_ and *mono*-cyclometalated complex **92**. Their electrochemical properties were compared with those of the corresponding coordination complex [Os(bpy)_3_](PF_6_)_2_ (**103**) and *mono*-cyclometalated complex [Os(phpy)(bpy)_2_]PF_6_ (**94**), (Figure 19). Each Os−C bond decreases the Os^II^/Os^III^ reduction potential by ca. 500 mV ranging from 888 mV for coordination compound **103** to −958 mV (vs. Ag/AgCl) for *tris*-cyclometalated complex **101** [79,80] (Figure 21).

**Figure 22:**
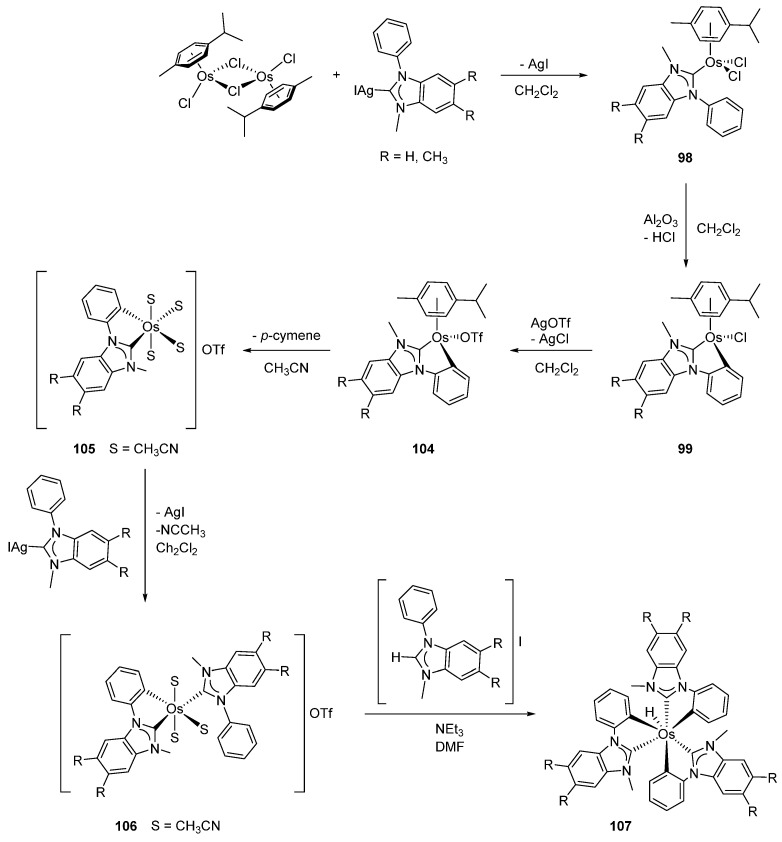
Synthesis *tris*-cyclometalated complex from the osmium dimer precursor.

Imidazolium complex **99** can be converted to a complex with four acetonitrile ligands **105**, which undergoes a second transmetalation with the organosilver compound to give **106**. The latter affords *tris*-cyclometalated complex **107** as shown in Figure 22 [77].

### 3.5. Other Precursors

**Figure 23:**
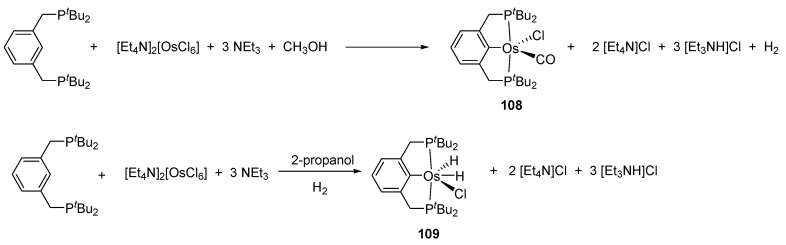
Synthesis of osmium P~C~P pincer using [Et_4_N]_2_[OsCl_6_].

Complexes [cation]_2_[OsX_6_] and [OsX_3_(tterpy)] (tterpy = 4′-(4-tolyl)-2,2′:6′,2″-terpyridine) were also used as osmacycle precursors. Complex [Et_4_N]_2_[OsCl_6_] reacts with 1,3-(CH_2_P*^t^*Bu_2_)_2_C_6_H_4_ in the presence of NEt_3_ affording 16e pincer complexes [OsCl(P~C~P)(CO)] (**108**) and [OsCl(H_2_)(P~C~P)] (**109**) [81]. It is suggested that Os^IV^ is first reduced to Os^II^ by methanol to produce CO. When the osmium precursor and the ligand are heated in 2-propanol, the solvent is not decarbonylated and dihydride complex **109** is obtained (Figure 23) [81].

**Figure 24:**
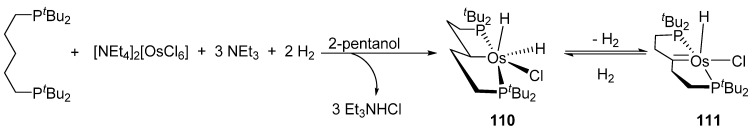
Osmium P~C~P pincer complex obtained by double C−H bond activation.

Complex [Et_4_N]_2_[OsCl_6_] reacts also with 1,5-*bis*(di-terbutylphosphino)pentane in the presence of NEt_3_ and H_2_ in 2-pentanol to give P~C~P alkyl complex **110**. A posterior thermal treatment initiates second C−H bond activation via α-elimination to give carbene complex **111** (Figure 24) [82].

**Figure 25:**
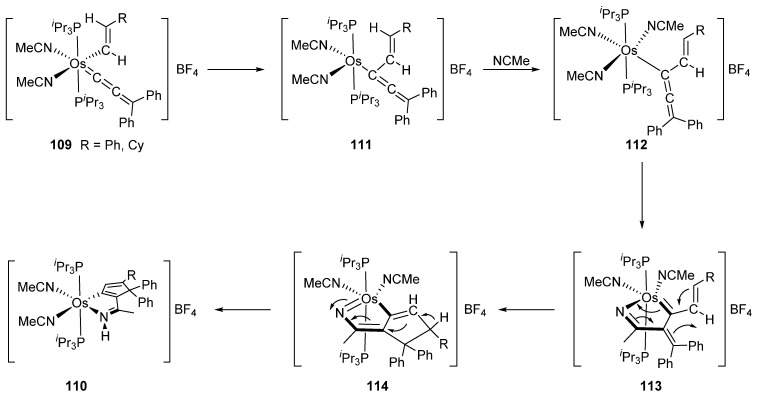
Synthesis of an osmacyclopentapyrrole complex from allenylidene derivative.

An interesting cycloosmation occurs within alkenyl–allenylidene–acetonitrile complex **109** which can form an osmacyclopentapyrrole at reflux in acetonitrile (Figure 25). The proposed mechanism is presented in Figure 25 [83].

**Figure 26:**
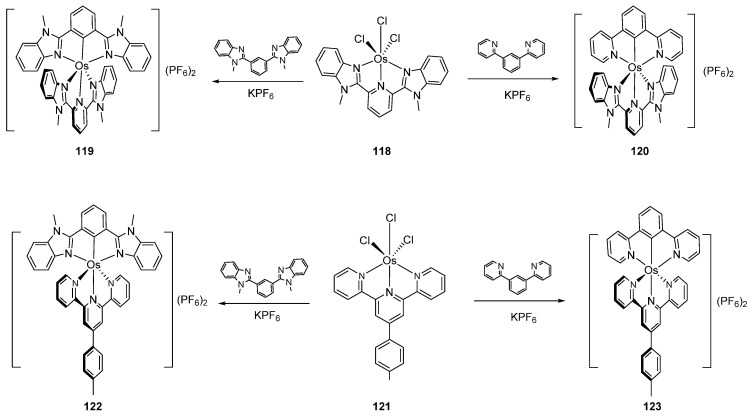
Synthesis of *bis*-pincer complexes.

Various Os^III^ bis(*N*-methylbenzimidazolyl)benzene or 1,3-di(pyridin-2-yl)benzene complexes were prepared from [OsCl_3_(Mebip)] (**118**) (Mebip = bis(*N*-methylbenzimidazolyl)pyridine) or [Os(ttpy)Cl_3_] (**121**) (ttpy = 4′-(4-tolyl)-2,2′:6′,2″-terpyridine) as shown in Figure 26 [84].

## 4. Representative Reactions of Osmacycles

### 4.1. Reactivity of Chelate and Pincer Complexes

**Figure 27:**
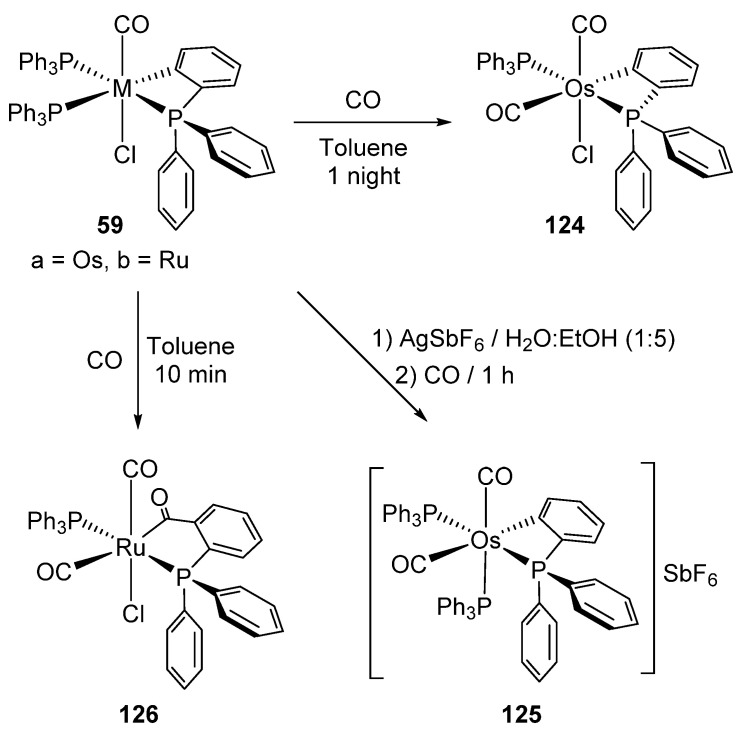
Reactions of four-membered metalacycles.

Ligand substitution in **59a** (see also Figure 14) yields neutral *mono*-osmacycle **124** [51]. On the other hand, ruthenium analogue **59b** displayed a different behavior since a parallel insertion of CO into the Ru−C bond in addition to ligand substitution accounts for the formation of **126** (Figure 27). In order to force the CO insertion into the Os−C bond, complex **59a** was treated with AgSbF_6_. However, the cationic species **125**, product of the substitution of the chloride ligand, was obtained [51].

**Figure 28:**
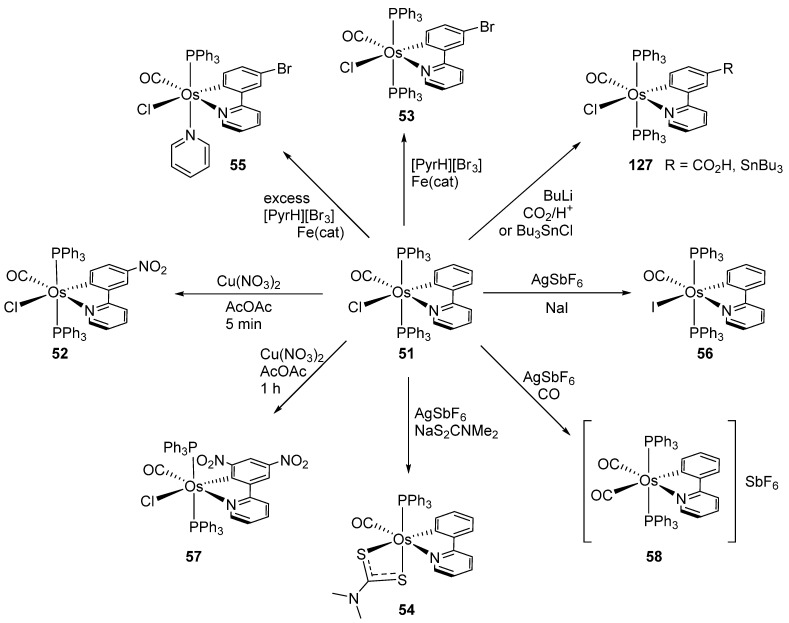
Electrophilic substitution reactions at the phenyl ring of cyclometalated complex **51**.

Reactions of 2-phenylpyridine osma(II)cycle **51** are summarized in Figure 28 [50]. The cyclometalated ligand is activated by Os^II^ towards electrophilic substitution allowing the introduction of diverse functional groups. Nitration occurs at both the 4- and 6-position of the phenyl ring, whereas bromination takes place at the 4-position only. Complex **51** reacts also with CO_2_/H^+^ or Bu_3_SnCl as shown in Figure 28 [50].

**Figure 29:**
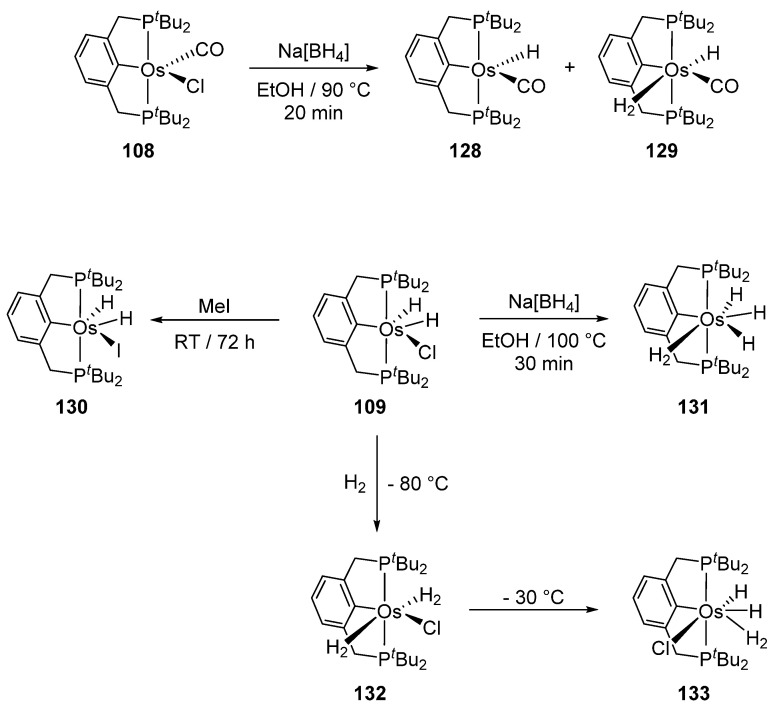
Reactivity of P~C~P pincer complexes **108** and **109**.

Gusev et al. studied the reactivity of P~C~P pincer compounds (Figure 29) [81]. Complex **108** reacts with NaBH_4_ and the mixture of two hydrides **128** and **129** is obtained. A chloride for iodide exchange takes place in **109** in the presence of MeI. The reaction of **109** with NaBH_4_ gives trihydride **131**. Complex **132** with two H_2_ molecules is also obtained in the presence of H_2_ at −80 °C; at higher temperatures one of the coordinated H_2_ undergoes oxidative addition to yield complex **133** [81].

**Figure 30:**
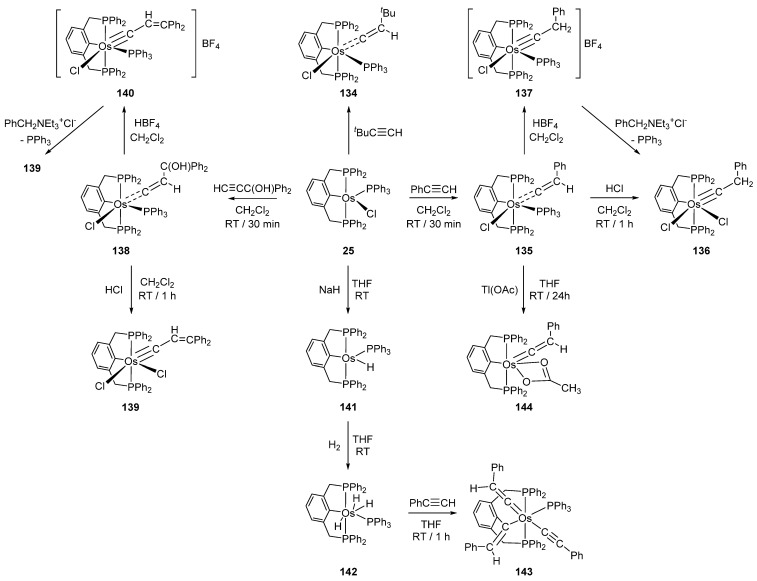
Reactions between osmium P~C~P pincer complex **25** with alkynes.

Reactions of P~C~P pincer complex **25** with a series of alkynes afford carbene and carbyne osmacycles (Figure 30) [40]. Complex **25** is also convertible to monohydride **141** and trihydride **142** in the presence of NaH (Figure 30). In an excess of phenylacetylene, **142** gives **143** with three incorporated phenylacetylene ligands. Remarkably, the coordination of all three is different including acetylide- and vinylidene-binding motifs. Vinylidene complex **135** reacts with Tl(OAc) to form **144**. Chloride is being replaced by acetate which acts as a bidentate ligand [85].

**Figure 31:**
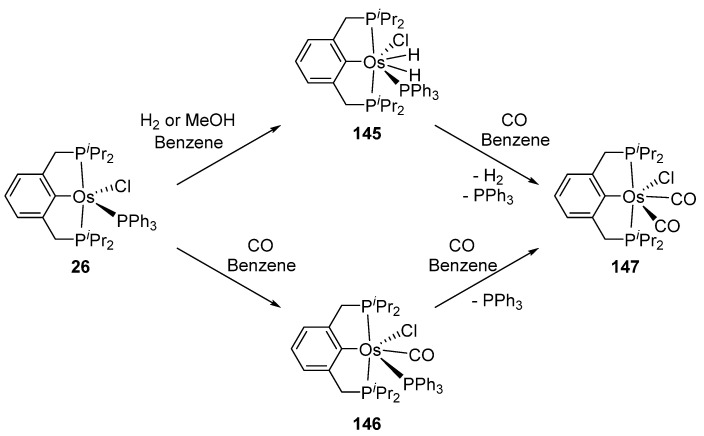
Reactions of P~C~P pincer complex **26** with H_2_ and CO.

Related P~C~P pincer complex **26** reacts with H_2_ giving rise to dihydride species **145**. Carbonyl derivatives **146** and **147** can also be prepared in the presence of CO (Figure 31) [41].

**Figure 32:**
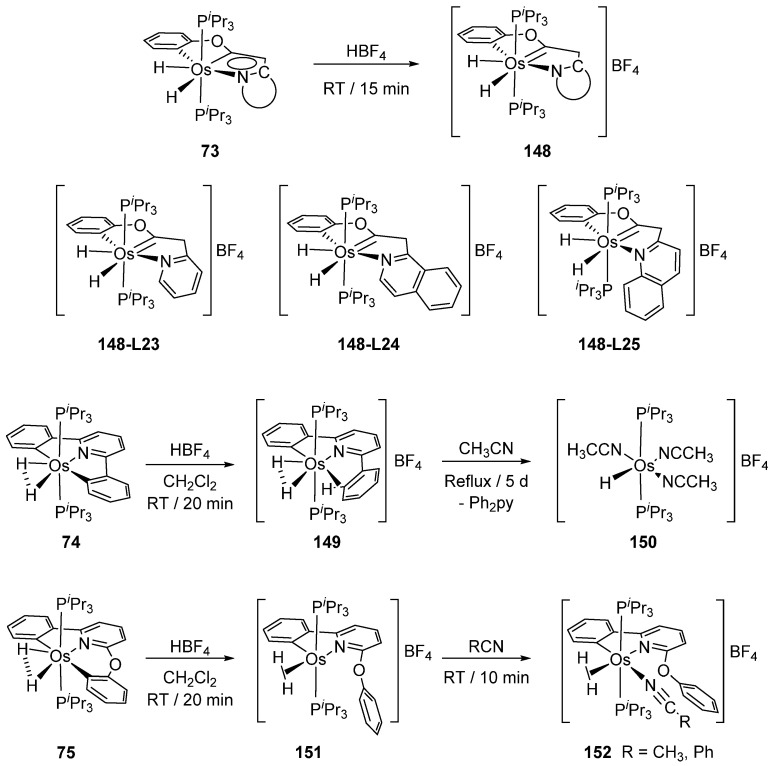
Protonation of the cyclometalated fragment by HBF_4_.

Dihydride [OsH_2_(P*^i^*Pr_3_)_2_(C~C′~N)] (**73**) reacts with HBF_4_ to yield [OsH_2_(P*^i^*Pr_3_)_2_(C~C’’~N)]BF_4_ (**148)** where the carbon in α position to the central carbon of the pincer ligand was protonated by the acidic medium [60]. Similarly, C~N~C’ pincer complexes **74** and **75** accept one proton at a metalated carbon to form cationic *mono*-cyclometalated complexes, which further react with nitriles as shown in Figure 32 [62].

### 4.2. Reactions of Hydride Complexes

**Figure 33:**
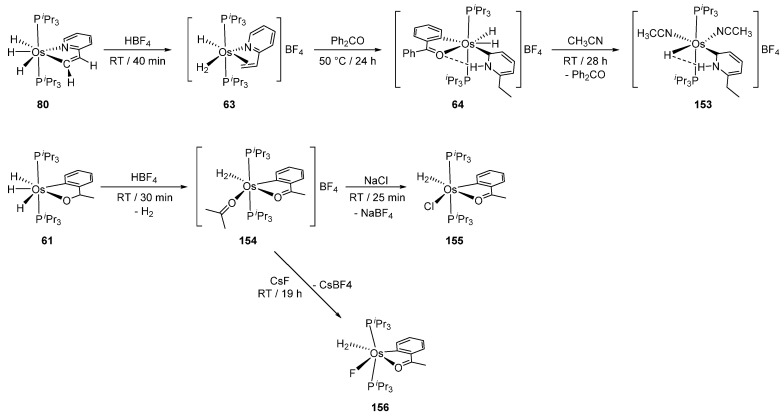
Reactions of hydride complexes with HBF_4_.

Hydrides in osmacycles promote reactions of adjacent ligands. For example, complex **80** reacts with HBF_4_ to give **63** with regenerated 2-vinylpyridine, the Os-trihydride unit being transformed into the Os-hydride-dihydrogen functionality. Subsequent treatment of **63** at 50 °C in the presence of Ph_2_CO induces the reduction of the double bond and formation of **64**. The elimination of benzophenone in acetonitrile at room temperature gives **153** [56]. In the case of trihydride complex **61** (Figure 33), addition of HBF_4_, however, affords dihydrogen derivative **154**, which is converted to cationic complexes (**155**, **156**) in the presence of NaCl or CsF, the cyclometalated fragment being unaffected [86].

### 4.3. Ligand Substitution in Osmacycles

**Figure 34:**
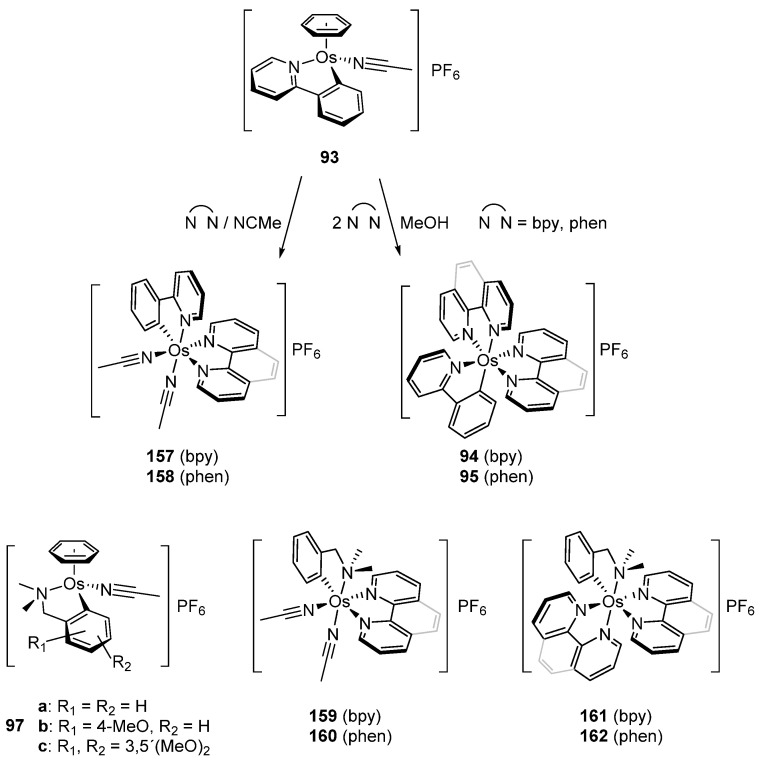
Synthetic routes to octahedral cyclometalated derivatives by ligand substitution starting from piano-stool complex **93**.

Piano-stool osmacyle **93** is a versatile precursor for a series of octahedral 2,2′-bipyridine, 1,10-phenanthroline and MeCN complexes (Figure 34). The substitutions are due to the lability of the coordinated benzene in polar solvents such as MeCN and MeOH. Cyclometalated *N,N*-dimethylbenzylamine derivatives **97** behave similarly affording complexes **159**–**162** [71,76,79,80].

**Figure 35:**
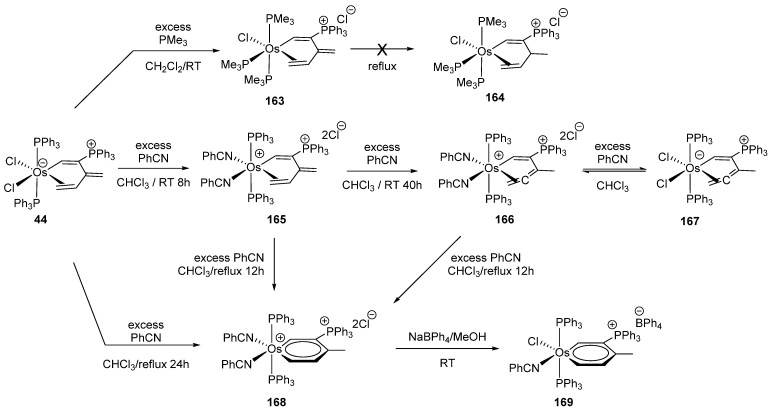
Studies of reactivity of complex **44**.

Complex **44** reacts with PMe_3_ and benzonitrile leading to cationic osmacycles. New species such as **167** with a η^2^-allene ligand are formed. Osmabenzene derivatives are also produced in many instances [87]. The reaction pathways of **44** are summarized in Figure 35.

**Figure 36:**
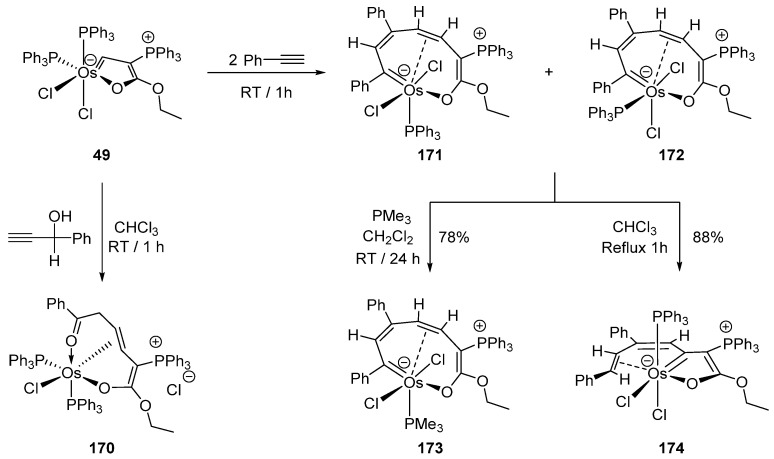
Ring-expansion reaction from a five-membered to a nine-membered osmacycle.

Interesting ring expansions of the cyclometalated fragment take place when **49** reacts with HC≡CCH(OH)Ph or PhC≡CH to give complexes **170**–**174** (Figure 36) [48].

## 5. Applications of Osmacycles

Osmacycles find applications in many areas including homogeneous catalysts; they are used as chemical sensors, luminescent materials, and anticancer agents. Relevant examples are summarized below.

### 5.1. Catalysis

**Figure 37:**
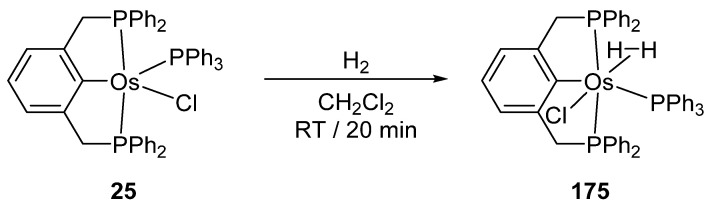
Coordination of dihydrogen to a pincer complex.

Although ruthenium and palladium cyclometalated complexes have been widely investigated in homogeneous catalysis [19,20,21,88,89,90], osmium analogues are not as widely used. Perhaps they have not yet demonstrated their potential. However, there are a few examples where osmacycles show good catalytic activity and look like promising candidates for new applications. In particular, binding of H_2_ to **25** affords **175** with η^2^-bound H_2_ (Figure 37) which might be a key intermediate in catalytic hydrogenation [91].

**Figure 38:**
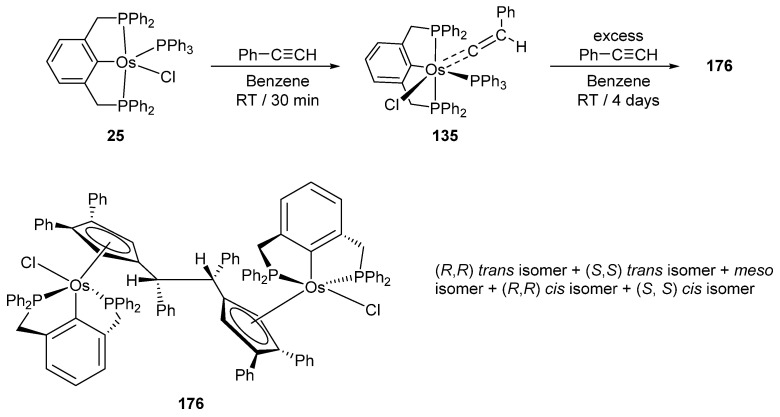
Hexamerization of phenylacetylene at osmium pincer complex.

Hexamerization of phenylacetylene at pincer complex **25** is a promising reaction which gives a mixture of isomers of diosmium complex **176** with a μ-1,2-bis(η^5^-cyclopentadienyl)-1,2-diphenylethane bridging ligand (Figure 38) [92].

**Figure 39:**
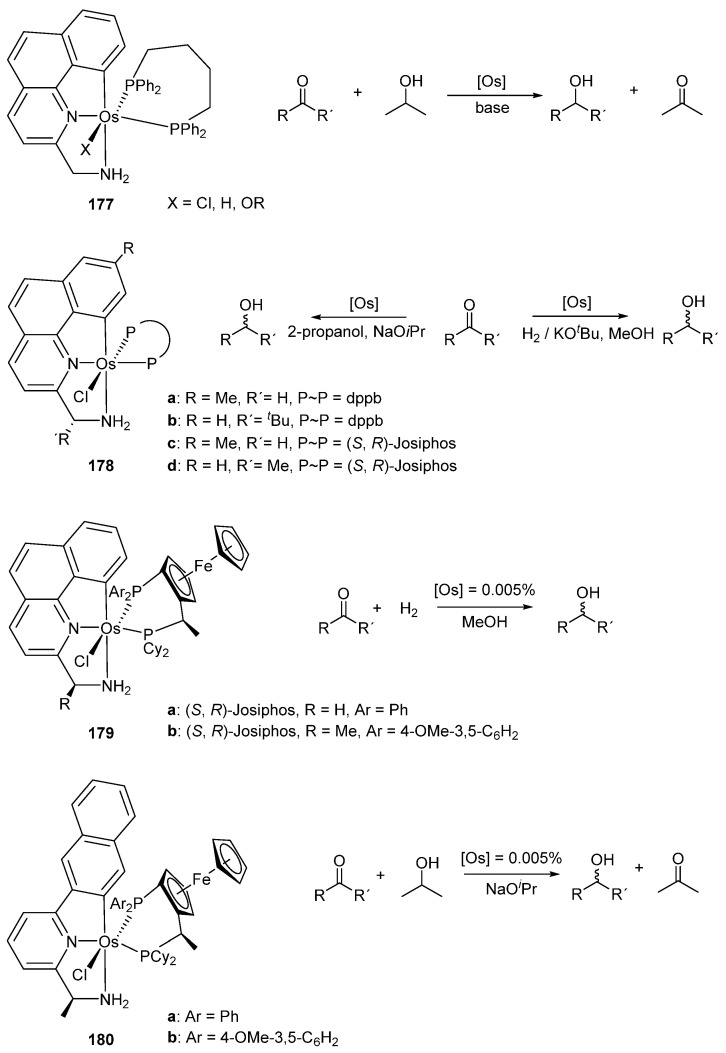
Hydrogenation reactions catalyzed by osmium pincer complexes.

Baratta et al. contributed significantly to the homogeneous catalysis by cyclometalated Ru and Os complexes. Benzo[*h*]quinoline C~N~N osmium pincers were tested as hydrogenation catalysts. Three osmium complexes [OsX(C~N~N)(dppb)] (**177**) (X = Cl, H, OR; dppb = 1,4-bis(diphenylphosphino)butane) were studied in catalytic hydrogen transfer from isopropanol to asymmetric ketones. Using a catalyst charge of 0.005% mol, conversions above 94% in less than 30 min with TOFs of 10^5^–10^6^ h^−1^ were achieved. The ruthenium analogues of **177** were slightly more active for the same process, with conversions above 97% within 10 min, presenting TOFs values in the order of 10^6^ h^−1^ [93]. Chiral ligands in **178** were tested for the asymmetric hydrogenation of prochiral ketones and conversions up to 92% with 74–94% enantiomeric excess (*ee*) were reported [94]. Structural modifications of the benzo[*h*]quinoline ligands gave two new series of chiral osmium complexes **179**. The complexes with a Josiphos ligand were used in the hydrogenation of acetophenone with conversions up to 97% with TOFs around 10^4^ h^−1^ and *ee* up to 86%. In the same work, the ruthenium analogue of **179a** was also studied. Conversions up to 95% with TOFs around 10^4^ h^−1^ and *ee* between 90 and 99% were obtained. However, in the case of ruthenium the reaction was performed at 40 °C instead of 70 °C for the osmium catalyst [95]. Different C~N~N pincer ligands were also used, keeping the Josiphos ligand system in complexes **180**. These were used for catalytic ketone reduction. Conversions were above 93% with TOFs 10^4^–10^5^ h^−1^ and *ee* between 90 and 99%. The ruthenium analogues were also obtained, and the catalytic activity for both metals was comparable [96]. The results are summarized in Figure 39.

**Figure 40:**
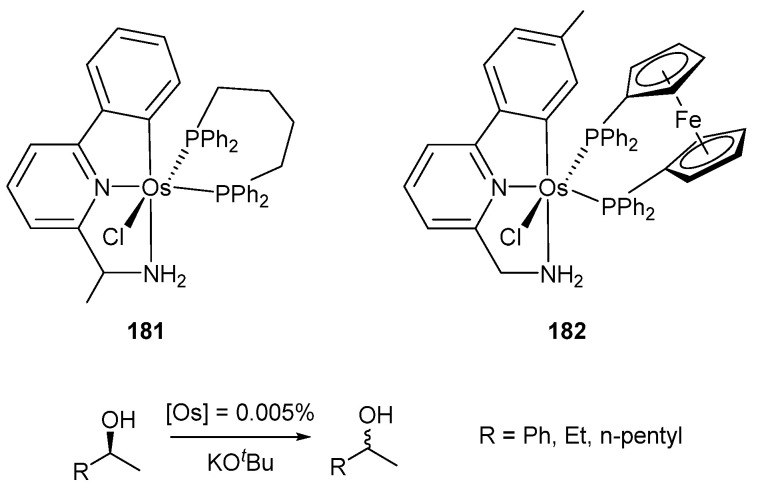
C~N~N pincer complexes used for racemization of chiral alcohols.

Osmium C~N~N pincer complexes **177** (X = Cl) and **178a** were employed in dehydrogenation of alcohols to form the corresponding ketones and H_2_. The dehydrogenation of α-tetralol using a catalyst charge of 0.4% mol was run with 36–44% conversions in 24 h. Their ruthenium analogues were also tested and were found to be more active, with conversions between 90–93% at the same reaction time [97]. The osmium C~N~N pincer complexes **181** and **182** were used for the racemization of alcohols with good results. In this case, a similar catalytic activity was observed for both ruthenium and osmium analogues (Figure 40) [98].

**Figure 41:**
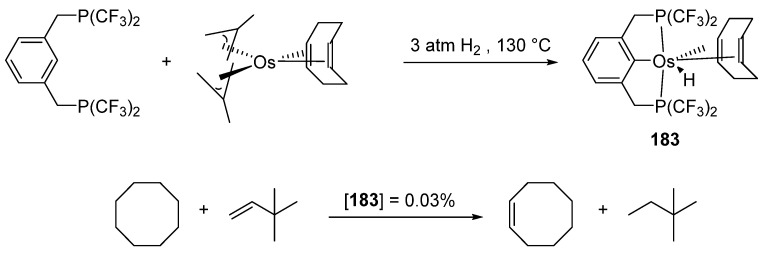
Alkane dehydrogenation catalyzed by a fluorinated P~C~P pincer complex.

Fluorinated pincer complex [OsH(^CF3^P~C~P)(cod)] (**183**) (Figure 41) obtained from [Os(cod)(η^3^-2-methylallyl)_2_] was tested in alkane dehydrogenation. The catalyst showed a longer lifetime than its ruthenium analogue. The dehydrogenation of cyclooctane in the presence of *tert*-butylethene as a hydrogen donor was carried out. A significant production of cyclooctene was detected within a few minutes at 200 °C [99].

Esteruelas’ group reported on the metalation of imidazolium salts using [OsH_6_(P*^i^*Pr_3_)_2_]. Stable compounds **185** and **187**, like possible intermediates in catalytic hydrogenation, were isolated (with yields from 83 to 89%) through the osmium reduction in the presence of HBF_4_. The authors also explored the substitution of the η^2^-bonded molecular hydrogen by a coordinating molecule like acetonitrile [100].

**Figure 42:**
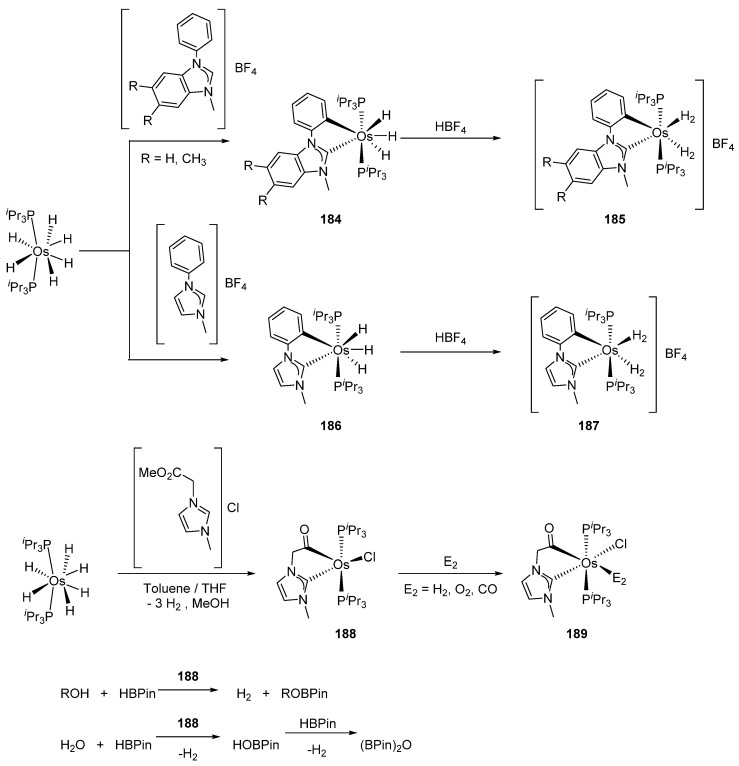
Examples of hydride complexes used in the alcoholysis and hydrolysis of pinacolborane.

**Figure 43:**
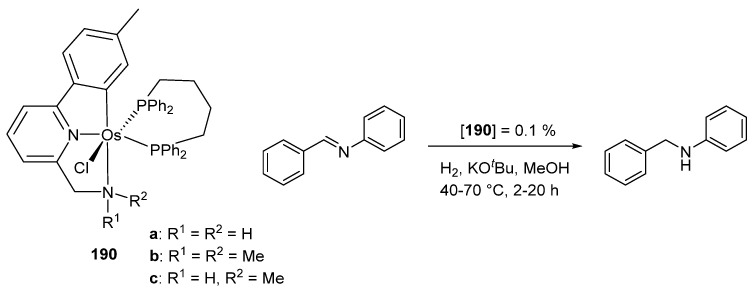
Hydrogenation of imines catalyzed by C~N~N pincer complexes.

The metalation of 1-(2-methoxy-2-oxoethyl)-3-methylimidazolium chloride afforded cyclometalated acyl complex **188** which coordinates dioxygen, dihydrogen, and carbon monoxide (Figure 42). Complex **188** was used as a catalyst for alcoholysis and hydrolysis of pinacolborane. Using a 2% mol catalyst charge for the alcoholysis reaction, TOFs between 62 and 3644 h^−1^ were obtained at 50% conversion. For the hydrolysis carried out with the same amount of catalyst, TOFs were 473–1648 h^−1^ at 50% [101]. The 2-(aminomethyl)pyridine-based complexes **190** were used in the imine-to-amine hydrogenation (Figure 43). Depending on the reaction conditions, conversions between 26 and 99% were observed. In this work, the ruthenium equivalents of **190** were also obtained. In general, under the same reaction conditions, the osmium complexes were found to be less active than the corresponding ruthenium derivatives [102].

**Figure 44:**
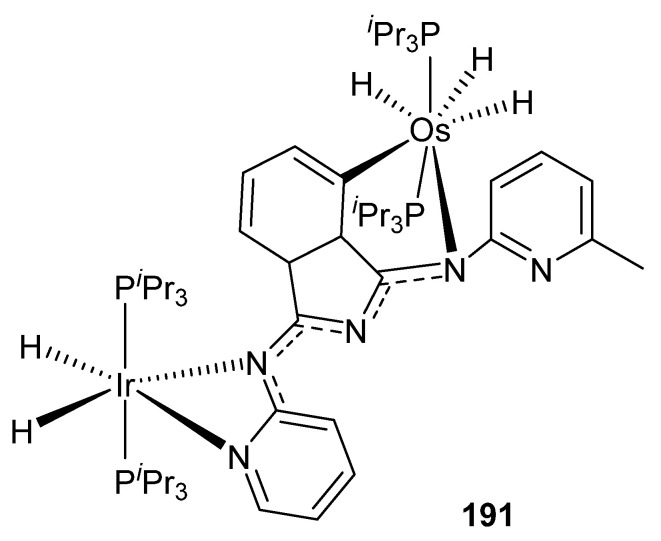
Heterobimetallic complex used in dehydrogenation of secondary alcohols.

Esteruelas et al. described the heterobinuclear iridium–osmium compound [(P*^i^*Pr_3_)_2_(H)_2_Ir{μ-(κ^2^-N_py_,N_imine_-BMePI-κ^2^-N_imine_,C^4^_iso_)}OsH_3_(P*^i^*Pr_3_)_2_] (**191**) incorporating 1,3-bis(6′-methylpyridyl-2′-imino)isoindoline (HBMePI) as a bridging ligand. This complex was tested in the base-free dehydrogenation of secondary alcohols using 7% mol of catalyst. Conversions up to 84% were obtained. Interestingly, heterobimetallic complex **191** proved to be more active than the corresponding monometallic components taken independently (Figure 44) [103].

**Figure 45:**
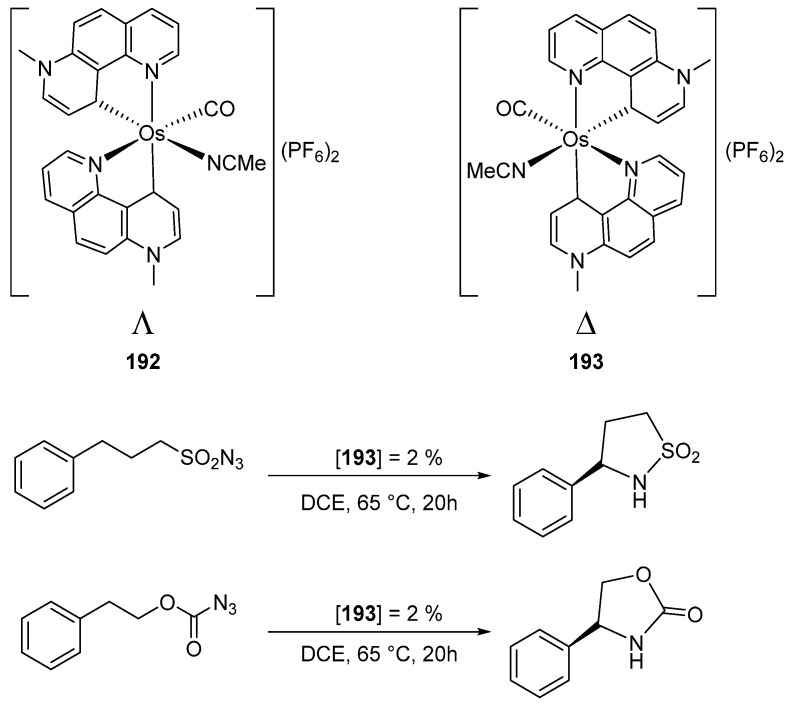
Asymmetric catalysis by cyclometalated osmium complexes with central chirality.

The resolution of a helical chiral octahedral osmium complex afforded optically pure Λ (**192**) and Δ (**193**) enantiomers (Figure 45). The Δ enantiomer was tested as a catalyst in two processes, viz. C(sp^3^)–H aminations of sulfonylazide and azidoformate. For the former, a product yield of 96% was observed using 2% mol of catalyst, with an enantiomeric ratio of 92:8 (Figure 45). Under similar conditions, a yield of 86% was registered in the case of azidoformate with the enantiomeric ratio of 89:11. This is the first example of an osmium complex with a central (helicoidal) chirality successfully used in asymmetric catalysis [104]. It is important to mention that Meggers’ group also developed highly effective enantioselective catalytic processes with metal-centered chiral octahedral ruthenium and rhodium complexes [105,106,107].

Octahedral cyclometalated 2-phenylpyridine Ru^II^ and Os^II^ complexes were investigated as catalysts for the atom transfer radical polymerization (ATRP). Polymerization of styrene was catalyzed by ruthenium complexes though the osmium counterparts were not able to mediate the polymerization and just traces of the polymer were observed [108].

### 5.2. Chemical Sensors and Biosensors

**Figure 46:**
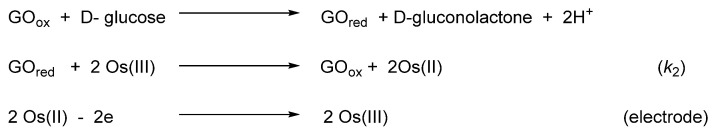
Electron transfer pathway between osmium shuttle and glucose oxidase (GO).

Oxidoreductases are enzymes that catalyze oxidative and reductive reactions. They are used in various amperometric biosensors, including glucometers [109]. Active sites of the enzymes do not usually exchange electrons with an electrode and therefore low-molecular-weight compounds are commonly used to move the electrons. Known as electron shuttles or mediators, they are often transition-metal complexes. In particular, cyclometalated ruthenium derivatives were successfully coupled with a number of oxidoreductases such as glucose oxidase, glucose and alcohol dehydrogenases, or peroxidases [110,111,112]. The M^II/III^ reduction potential is an essential feature for optimal performance of biosensors [113], and since the potentials are tunable as it is illustrated in Inset to Figure 21 [80], the corresponding osmium-based mediators were exploited. In particular, a series of octahedral osmium(II) complexes of the general formula [Os(N~C)(N~N)(MeCN)_2_]PF_6_ (**159**, **161**) and [Os(N~C)(N~N)_2_]PF_6_ (**94**, **95**, **162**, **163**) were investigated, where C~N is cyclometalated 2-phenylpyridine or *N,N*-dimethylbenzylamine and the N~N ligands are 2,2′-bipyridines or 1,10-phenanthrolines. The complexes were evaluated as electron shuttles with glucose oxidase (GO) for potential applications in amperometric enzymatic glucose sensors. All complexes showed extremely fast electron transfer with the enzyme, with the second-order rate constants *k*_2_ for bimolecular oxidation of reduced glucose oxidase in the range of (0.67 − 2.90) × 10^6^ M^−1^ s^−1^ (Figure 46 and Table 3) [76].

**Figure 47:**
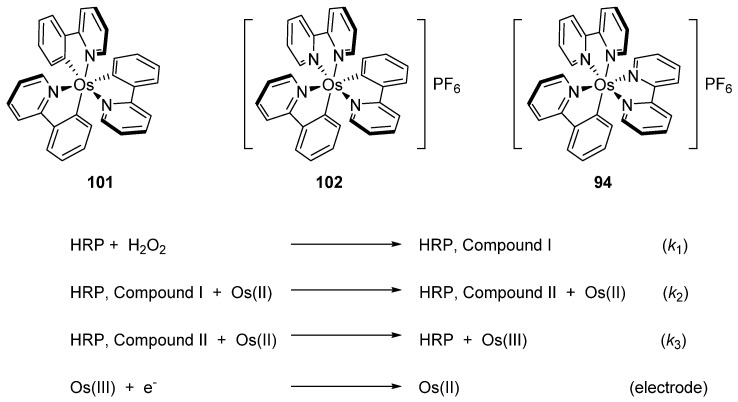
*Mono*-, *bis*-, and *tris*-cyclometalated complexes used as electron shuttles and electron transfer pathway with horseradish peroxidase (HRP).

Discussed above *mono-* [Os(phpy)(bpy)_2_]PF_6_ (**94**), *bis-* [Os(phpy)_2_(bpy)]PF_6_ (**102**) and *tris*-cyclometalated [Os(phpy)_3_] (**101**) derivatives turned out to be attractive objects for electrochemical studies. Using related coordination complex [Os(bpy)_3_](PF_6_)_2_ (**103**), it was shown that the Os^II^/Os^III^ reduction potential significantly decreases as the number of Os−C bonds increases. Rates of electron transfer between the osmium center and the active sites of oxidoreductases including horseradish peroxidase (HRP) were investigated (Figure 47). Cyclic voltammetry was used to estimate the rate constants. In the case of HRP, the results showed that the rate constants *k*_2_ are higher than *k*_3_ for all compounds, i.e., electrons move faster from Os^II^ to compound I than to compound II (see Figure 47). The value of both rate constants increases in the series [Os(bpy)_3_](PF_6_)_2_ < [Os(phpy)(bpy)_2_)]PF_6_ (**94**) < [Os(phpy)_2_(bpy)]PF_6_ (**102**) and then decline for the *tris*-cyclometalated complex (**101**). It should be mentioned that all complexes are very reactive. The Monte Carlo docking simulations helped to evaluate how the complexes approach the active site of HRP [80].

**Figure 48:**
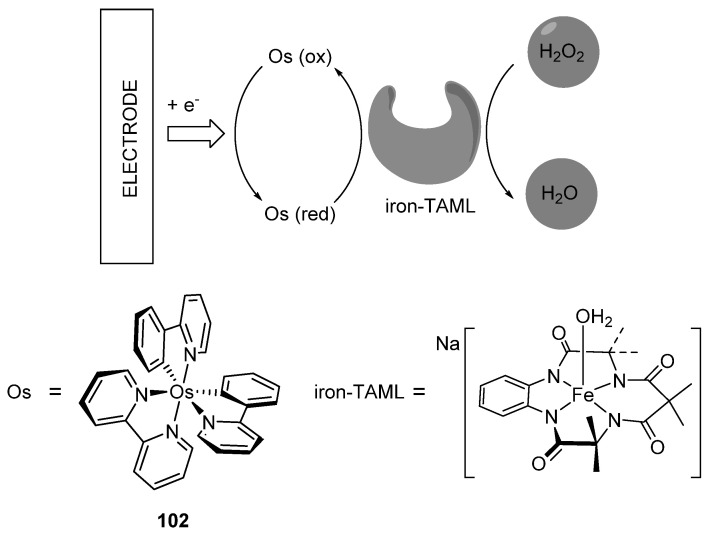
Conceptual principle for a sensor using an iron(III)-TAML catalyst and the *bis*- osmacycle as a mediator.

*Bis*-cyclometalated complex [Os(phpy)_2_(bpy)]PF_6_ (**102**) was tested as a mediator in a prototype of an amperometric biosensor to quantify hydrogen peroxide (Figure 48) An iron(III)-TAML (TAML = *t*etra*a*mido *m*acrocyclic *l*igand) catalyst used as an alternative to peroxidase enzymes was immobilized on an electrode surface with the osmium mediator. The activity and sensitivity of such a device were similar to those of a biosensor based on HRP enzyme [114].

### 5.3. Electronic Properties and Photophysics

Due to a unique combination of spectroscopic, photophysical, photochemical and electrochemical properties, octahedral Ru^II^ and Os^II^ polypyridyl complexes have been extensively studied in various fields such as photochemical conversion of solar energy, photocatalysis and in molecular electronic devices. Their properties can be modulated by modifying the ligand structures and by introducing ancillary ligands [115,116]. The major goal is to enhance excited state lifetimes of the compounds at room temperature by increasing the energy gap between the radiative ^3^MLCT and quenching ^3^MC states by destabilizing the ^3^MC state using cyclometalated ligands [117,118]. However, the enhanced spin-orbit coupling of the third-row elements when compared to the second-row elements usually makes optoelectronic properties of osmium complexes notably different from those of ruthenium derivatives [119]. Nevertheless, as we discuss in this section, switching from ruthenium to osmium has proven to be successful in generating promising materials.

**Figure 49:**
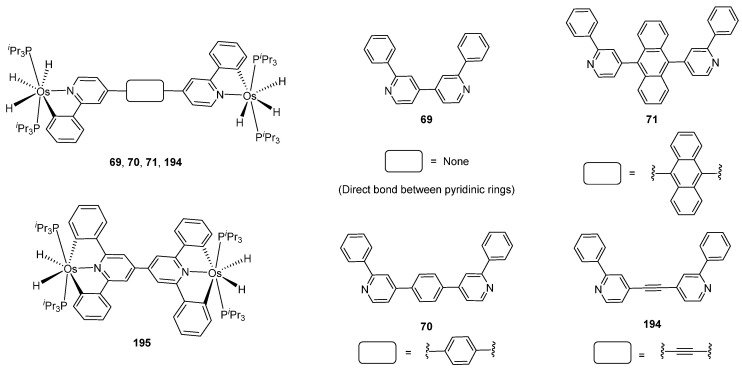
Bimetallic luminescent complexes.

Sierra et al. synthesized dinuclear cyclometalated trihydride osmium(IV) complexes (**69**–**71**, **194**, **195**) (Figure 49) which were studied by cyclic voltammetry. Two quasi-reversible processes corresponding to the Os^IV^/Os^V^ and Os^V^/Os^VI^ redox features were observed. The Os^IV^ complexes showed emission around 360 nm, with quantum yields being between 0.005 and 0.020. The excitation spectra suggested that a ligand-centered transition is at the origin of the emission. Moreover, spectrochemical studies showed that the emission spectra remained very similar upon Os^IV^ to Os^V^ oxidation at 0.05 V vs. Fc^+^/Fc, since only a slight increase in intensity was observed, with quantum yields from 0.013 to 0.026. Nevertheless, when a potential of 1.10 V was applied to carry out the oxidation of Os^V^ to Os^VI^, a bathochromic shift, from around 360 to around 400 nm, was observed along with an increase in the luminescence intensity with quantum yields around 0.030–0.110. DFT calculations suggest that the photophysical properties vary due to the conversion of trihydride species to monohydride-dihydrogen derivatives during sequential oxidation processes [59].

**Figure 50:**
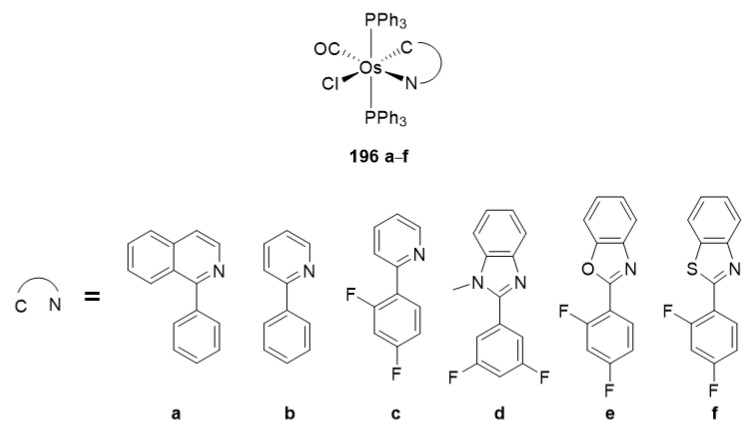
Luminescent [OsCl(N~C)(PPh_3_)_2_(CO)] complexes.

Kapturkiewicz et al. prepared the series of cationic osmium complexes **196** of the general formula [OsCl(N~C)(PPh_3_)_2_(CO)] (N~C is a cyclometalated substituted 2-phenylpyrididine) and measured their UV-visible absorption and emission spectra (Figure 50). The complexes showed emission at 455–532 nm attributed to ^3^MLCT transitions. Importantly, only the solids emitted light at room temperature; the compounds in solution were emissive only at a low temperature (77 K). The emission lifetimes are relatively long, between 10 and 20 μs [120].

*Bis*-(P~C~P) pincer osmium complex **39** (Figure 10) has a strong absorption at 250 nm attributed to the ligand π→π* transitions. The emission at 546 nm in a dilute solution of 2-MeTHF at 77 K with a quantum yield of 0.6 and a lifetime of 8.0 μs suggests phosphorescence. The solid complex is also luminescent at room temperature at 556 nm, with a quantum yield of 0.03 and a lifetime of 0.3 μs. The electronic structure of the complex and related transitions were explored by DFT [45].

**Figure 51:**
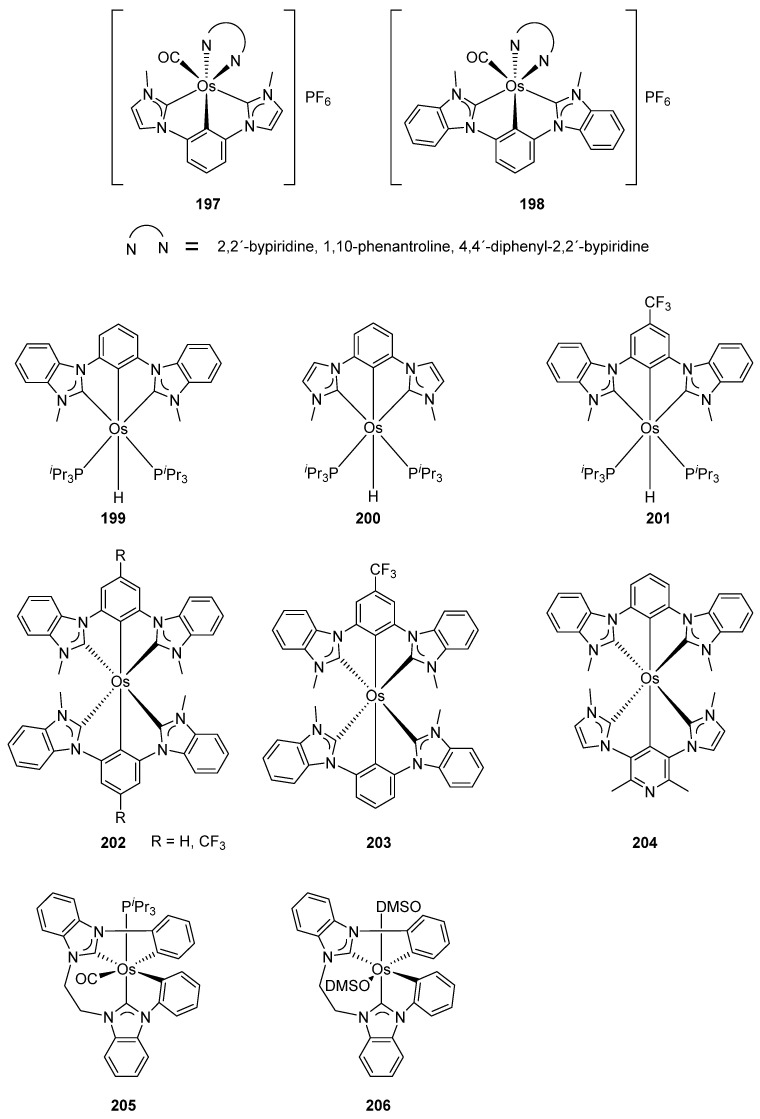
Luminescent carbene-based pincer complexes.

Carbene pincer ruthenium and iridium compounds display remarkable luminescent properties [121]. Related osmium C~C’~C complexes **197** and **198** were obtained by reacting [OsCl_4_(N~N)] precursors (N~N = 2,2′-bypiridine, 1,10-phenanthroline, 4,4′-diphenyl-2,2′-bypiridine) with *bis*-imidazolium or *bis*-benzimidazolium hexafluorophosphates in refluxing ethylene glycol as a source of the CO ligand (Figure 51). The UV-visible absorption data showed intense and high-energy absorption at λ lower than 330 nm and a less intense band at λ > 330 nm. The complexes are also emissive upon photoexcitation with emission maxima in the red region (674−731 nm). The quantum yields reported were between 10^−4^ and 10^−2^ and emission lifetimes in the order of 10^−1^ μs. The photophysical parameters are sensitive to the modification of N~N and C~C’~C parts, suggesting that the emissive-excited state should involve both fragments and the emission occurs due to the energy dissipation of d(Os)→π*[N~N] MLCT transitions [122].

The photophysical studies of the C~C’~C *mono-* and *bis*-pincer compounds (**199**–**204**) in Figure 51 revealed an intense absorption between 341 and 363 nm along with a less intense band between 396 and 405 nm. TD-DFT calculations allowed to attribute the high energy bands to interligand charge-transfer transitions and the low energy bands to metal-to-ligand charge-transfer processes. Particularly, the *bis*-pincer complexes **202**–**204** exhibited emission in the blue-green region (475–578 nm), both in the solid state at room temperature and in toluene solution (at room temperature and 77 K) with lifetimes in the range of 10–29 μs. The best quantum yield is observed for complex **202** (R = CF_3_) with a value of 0.62. This last complex **202** (R = CF_3_), with emission in the blue region, was chosen for the fabrication of a model OLED device, obtaining promising results [123].

The osmium complexes **205** and **206** with a tetradentate C~C~C~C ligand (C_phenyl_~C_carbene_~C_carbene_~C_phenyl_) in Figure 51 can be viewed as *bis*-cyclometalated compounds. The complexes with two coordinated DMSO ligands provide options for their replacement by bidentate diphosphines or dienes. Complexes bearing 1,2-bis(diphenylphosphino)benzene emit light upon photoexcitation in the solid state at room temperature and in solution of 2-MeTHF at 77 K. The bands between 630 and 549 nm were attributed to the π-π* HOMO (Os and NHC fragment) to LUMO (diphenylphosphino) and metal-to-ligand (osmium to phosphino ligand) charge-transfer transitions. In addition, the absorption spectra contained an intense band at 330 nm and a less intense band at 404 nm attributed to metal-to-ligand charge-transfer transitions [124].

**Figure 52:**
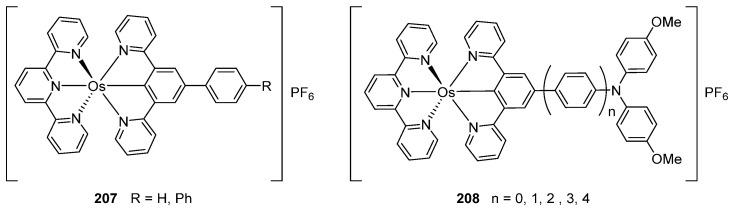
*Bis*-tridentate cyclometalated osmium complexes studied as molecular wires.

*Bis*-tridentate cyclometalated complexes [Os(N~N~N)(N~C~N)]PF_6_ (**207**, **208**) have been tested as molecular wires, and compounds **208**, in which the N~C~N ligand with a triarylamine substituent is separated from the metal center by an oligophenyl bridge, were of special interest (Figure 52). This is due to the presence of two electrochemically active centers, the metal and the triarylamine substituent which can undergo a N^•+^/N^0^ process (neutral nitrogen to positive radical nitrogen). These molecular wires show two anodic redox features between +0.1 and +0.9 V vs. Ag/AgCl, the first one assigned to the Os^II^/Os^III^ process and the second one to the N^•+^/N^0^ process, and an electron transfer from the neutral amine to the Os^III^ center was observed in the one-electron oxidized form [Os^III^-N] [125].

Esteruelas et al. studied the luminescent properties of C~C~N pincer complexes derived from 2-azetidones (see Table 2). At room temperature, complex (**73**-**L22**) showed yellow emission in the solid state at 571 nm (lifetime 1.1 μs) and at 562 nm in a toluene solution (lifetime 2.0 μs), as well as green emission at 77K at 540 nm (lifetime 6.6 μs) [60]. Complexes **74** and **75** in Table 2 are phosphorescent upon photoexcitation in a poly(methyl methacrylate) (PMMA) film and in 2-MeTHF at room temperature and 77 K. Both compounds emit in the yellow region. The 2,6-diphenylpyridine complex **74** is more efficient, the quantum yields being 0.28 (film) and 0.56 (solution). The introduction of oxygen between the pyridine and phenyl ring in **75** lowered the emission (quantum yields below 0.10). Curiously, the iridium analogue of **75** showed the opposite trend [126].

**Figure 53:**
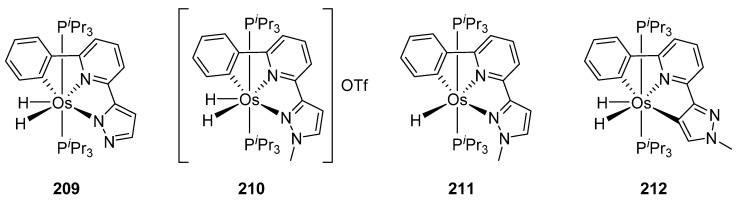
Phosphorescent C~N~N and C~N~C osmium pincer complexes.

The absorption spectra of C~N~N and C~N~C pincer complexes **209**–**212** in Figure 53 are similar. They present two main bands between 279 and 317 nm and between 364 and 394 nm. TD-DFT calculations allowed to assign the high energy absorptions to ligand-centered transitions while the low energy absorptions were assigned to metal-to-ligand charge-transfer processes. The compounds were emissive upon photoexcitation in the solid state at room temperature and in 2-MeTHF solution both at room temperature and at 77 K. The use of pincer ligands allowed to obtain more rigid structures reducing both the energy dissipation and the difference between the excited state and the ground state structures. As a consequence, enhanced quantum yields in the range of 0.08–0.59 were obtained, lifetimes being 1.5−5.0 μs in the solid state and 0.9–5.8 μs in solution [127].

**Figure 54:**
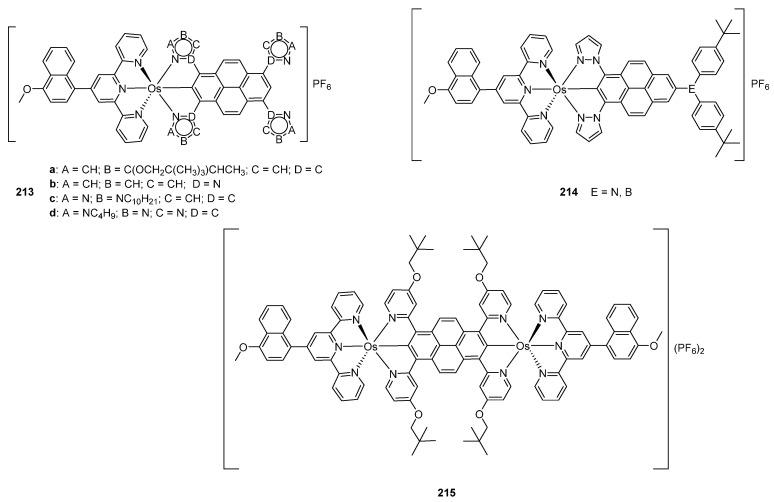
Luminescent *bis*-pincer complexes with cyclometalated pyrene-type ligands.

The electronic structure of osmium *bis*-pincer complexes **213** with N~C~N cyclometalated pyrene ligands (Figure 54) was explored by DFT and TD-DFT. It was found that HOMO orbitals are mainly osmium in character. The LUMO orbitals were largely associated with ligand contributions. There are two intense bands absorption spectra between 282 and 372 nm assigned to intraligand transition from the pyrene and terpyridine fragments. Two other intense bands between 410 and 447 nm were associated with intraligand charge-transfer (ILCT) transitions. The bands observed between 513 and 556 nm were due to MLCT transitions [128]. These experimental results launched a theoretical study which helped to optimize photophysical features of the compounds. It was concluded that compounds **214** in Figure 54 should display optimal properties [129].

DFT calculations performed for bimetallic complex **215** shown in Figure 54 suggested that the HOMO orbital is contributed by pyrene and metal centers. The LUMO orbital has contributions from the pyrene moiety and pyridine rings. Two intense bands observed between 457 and 507 nm were assigned to ligand-to-ligand charge-transfer transitions. An absorption band between 800 and 1000 nm was assigned to metal-to-ligand (pyrene) charge-transfer transitions. An electronic coupling between the neighboring osmium centers affects cyclic voltammograms of osmium compounds and brings about two reversible Os^II^/Os^III^ redox features overlapping at −0.15 V (vs. Fc/Fc^+^). A similar behavior is observed for ruthenium but not for iridium complex [130].

### 5.4. Anticancer and Biological Properties

Transition metal derivatives have been extensively studied in medicinal chemistry, often with emphasis on their anticancer activity. Organometallic complexes are currently receiving special attention [131,132,133,134,135,136,137]. The leadership belongs to ruthenium compounds including the clinical studies of metal-based anticancer drugs [138,139,140,141]. Omae published a comprehensive review on anticancer properties of cyclometalated complexes with a few osmium examples [132]. Pfeffer and Gaiddon dedicated a review to ruthenacycles [142]. Examples of a few osmacycles were included in the recent review by Zhang devoted to biological properties of osmium compounds [131]. Here, we show the most recent examples of osmium cyclometalated complexes used in biological assays.

**Figure 55:**
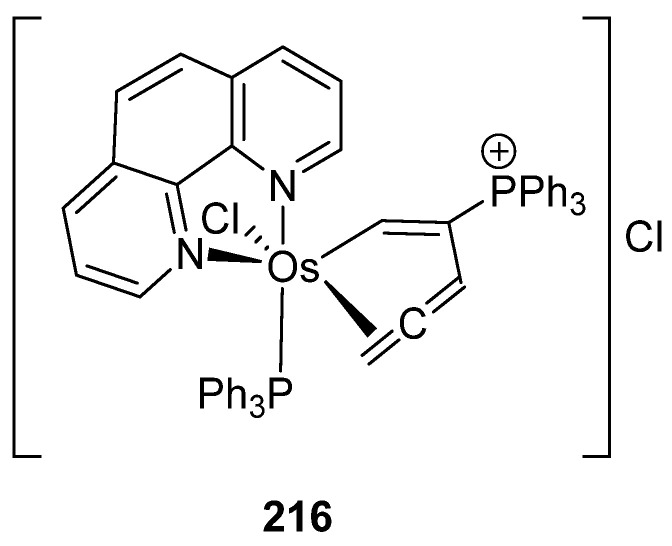
η^2^-Allene complex with promising cytotoxic activity.

Meggers and Xia compared the anticancer activity of a series of osmium derivatives, including complexes **43**, **49**, **164**, **174**, a new η^2^-allene compound (**216**, Figure 55), and other non-cyclometalated complexes. The cytotoxic activity in HeLa cancer cells was determined first by the evaluation of the half-maximum effective concentration (EC_50_) at which viability of the cells is reduced to 50% after 24 h incubation. Observed values for EC_50_ were between 1 and 30 μM. The best results were obtained with allene complex **216** which exhibited cytotoxic activity with EC_50_ = 1 μM. Because of this and its high stability, studies of this complex were prioritized. The compound was tested against Burkitt-like lymphoma (BJAB), showing an inhibition of the proliferation at concentrations lower than 1 μM. Another assay revealed that the compound induces apoptosis in BJAB cells by DNA fragmentation. The results showed that even a 0.3 μM concentration, **216** induced DNA fragmentation in 25% of the cells, with the highest DNA fragmentation observed in 75% of the cells at 5 μM. In addition, other experiments showed that apoptosis occurs through the intrinsic mitochondrial pathway. The compound was also tested in leukemia cells (Nalm6), Vcr-resistant NAlm6 cells and Dau-resistant Nalm6 cells (the last two are drug-resistant leukemia cells) showing a lower activity, with EC_50_ higher than 1 μM, but with effective activity at 5μM [143].

**Figure 56:**
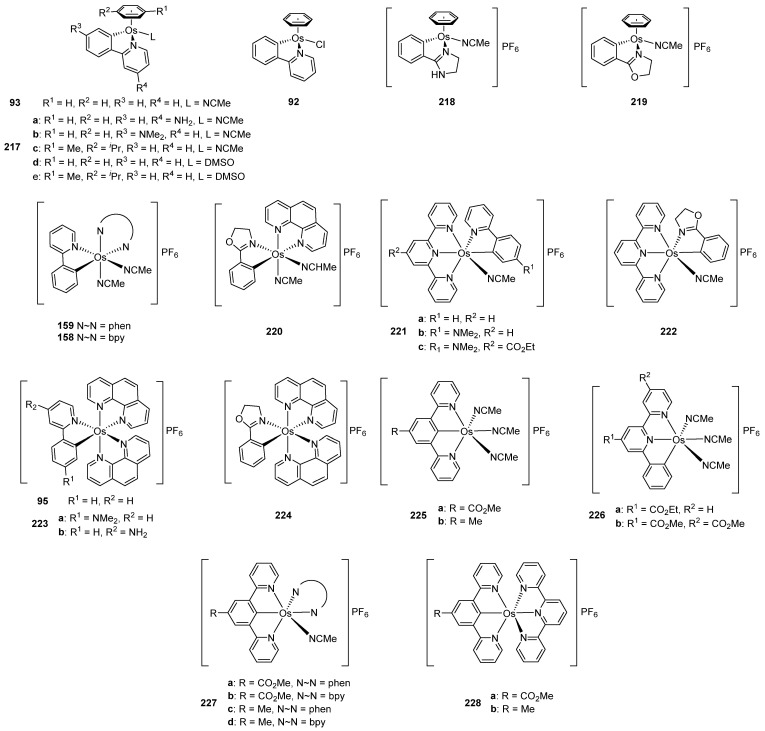
Series of cyclometalated osmium complexes tested in the in vitro cell growth inhibition in A172 glioblastoma cell line.

The successful use of ruthenacycles and osmacycles as efficient electron shuttles for oxidoreductase enzymes described in Section 5.2 prompted the investigation of the potential use of these compounds for biological applications. Gaiddon and Pfeffer evaluated the cytotoxic properties of such compounds on series of human cancer cell lines. An exhaustive work was published where a series of osmium cyclometalated compounds was tested in in vitro cell growth inhibition in A172 glioblastoma cell line. The structures of the compounds studied are summarized in Figure 56. First results with the piano-stool complexes (**92**, **93**, **217**–**219**) showed moderate cytotoxic activity with IC_50_ values of 10−100 μM. The octahedral complexes with bidentate cyclometalated ligands (**94**, **158**, **159**, **220**–**224**) exhibited very promising results with IC_50_ values in the nanomolar range. Pincer complexes (**225**, **226**) showed very poor cytotoxic activity, with IC_50_ values 21−375 μM, which could be attributed to the presence of three labile acetonitrile ligands. Interestingly, the pincer complexes with polypyridine ligands (**227**, **228**) showed very good results with IC_50_ values between 0.3 and 4.1 μM. There was no correlation between the cytotoxicity and structure. However, the reduction potentials were measured for all complexes and the results showed that the compounds with the potentials between 0.3 and 0.6 V (vs. SCE) displayed the lowest IC_50_ values. The lipophilicity was also determined and the complexes with the highest activity showed log(P*_o/w_*) values around 2. These results allowed to hypothesize that the compounds could strongly modify the metabolism of the cells by interactions with oxidoreductases and this would partially explain the relationship between the activity and the reduction potentials [144]. Another important target of such studies is to discover molecules that can overcome drug resistance induced by cisplatin. Recently, the mechanisms governing the variability in the cytotoxicity of two ruthenium cyclometalated compounds and their osmium equivalents were studied (Figure 34, complexes **95** and **159**). Their anticancer properties in vitro and in vivo were first evaluated and genes involved in their sensibility/resistance were identified by correlating their cytotoxicity with transcriptomic data of 60 cancer cell lines. Docking and functional studies demonstrated that inhibition of known resistance mechanisms, ABCB1 export and EGFR expression, allowed to improve the activity of cyclometalated complexes. Interestingly, switching from ruthenium to osmium favored the cytotoxicity while reducing the sensibility to the ABCB1 export mechanism [145].

**Figure 57:**
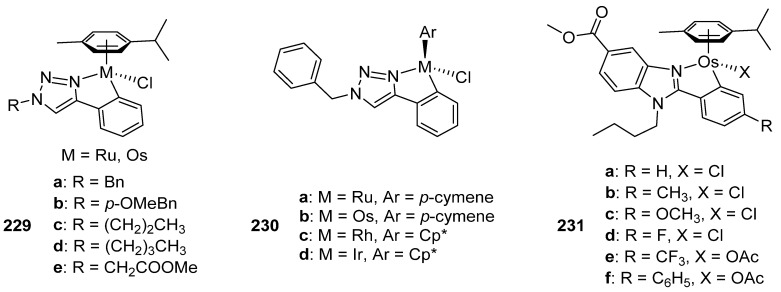
Complexes tested against various human cancer cell lines.

Kandioller developed the coordination of a series of 4-phenyl-1,2,3-triazole-based ligands to ruthenium and osmium to prepare complexes **229** in Figure 57. Their cytotoxic activity was evaluated on three cancer cell lines: non-small-cell lung cancer (A549), colon adenocarcinoma (SW480) and human ovarian carcinoma (CHI/PA-1). The free ligands did not show cytotoxic activity within the studied concentrations range. However, the ruthenium derivatives bearing ligands **a** and **b** showed significant cytotoxicity with IC_50_ values around 13 μM in A549, 7 μM in SW480 and 4 μM in CHI/PA-1. Osmium counterparts displayed an even higher activity, ligands **a** and **b** being also the most active, with IC_50_ around 6 μM for A549, 3.6 μM for SW480, and 1 μM for CHI/PA-1 cells. The topoisomerase IIα inhibition was investigated but no compound was capable of inhibiting its function, indicating that the mechanism of action does not follow this pathway. The cell cycle distribution in SW480 in response to treatment with the complexes was determined and only the osmium derivatives exhibited elimination of the S phase fraction, information relevant in order to understand the mechanism of action [146].

Using a triazole-based ligand, Makhubela prepared a series of half-sandwich ruthenium, rhodium, iridium and osmium complexes (**230**), Figure 57. The complexes were tested on different cancer cell lines (leukemia MT4, cervical cancer HeLa, kidney adenocarcinoma HEK293 and lung cancer A549), and the CC_50_ (cytotoxic concentration with 50% of the maximum decrease in cell viability) was calculated. The ruthenium derivative (**230a**) exhibited a moderate activity against A549, but the osmium compound (**230b**) was much more active with a CC_50_ value of 6.4 μM, comparable to cisplatin used as reference. However, its activity was very modest against the other cancer cell lines. Interestingly, the rhodium and iridium derivatives were more effective against all cancer cell lines when compared to their ruthenium and osmium analogues [147].

Benzimidazole-based ligands were used by Ruiz to prepare the series of new osmium complexes **231**, Figure 57. Their antiproliferative activity was tested in six different human cancer cell lines (including cisplatin-resistant cell lines), as well as non-tumorigenic human endothelial hybrid cells and Buffalo green monkey cells. All osmium compounds showed a higher cytotoxic activity than cisplatin in all cancer cell lines, with IC_50_ values lower than 10 μM. The cytotoxicity against the non-cancer cells was very similar to cisplatin. The cellular concentrations of both metals were measured in A2780 cells and the results showed that the osmium cellular uptake is ten times higher than for platinum. Other studies revealed that the compounds caused a decrease of the ROS levels and, as a result, an affectation in the G0/G1 phase in the cell cycle is observed [148].

**Figure 58:**
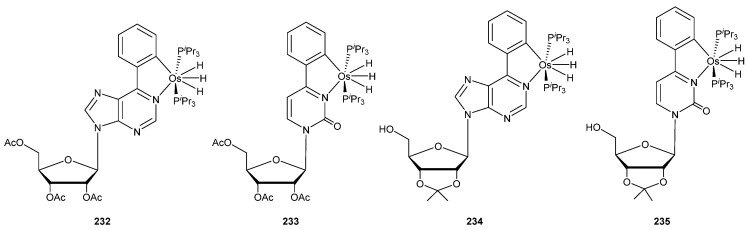
Osmium complexes using nucleosides as cyclometalating ligands.

In a very recent work, Gómez-Gallego, Sierra and Esteruelas explored the reaction between osmium precursor [OsH_6_(P*^i^*Pr_3_)_2_] and nucleosides. Cyclometalated complexes **232**–**235** were obtained in good yields (Figure 58). The authors mentioned that this methodology could be applied to the functionalization of oligonucleotides with promising potential biological applications [149].

Many more biological applications of osmium-based complexes remain to be discovered and exploited. For instance, the unique photophysical properties of osmacycles (see Section 5.3) will undoubtedly open the field for further research on their use as PDT agents. On the other hand, deeper in vivo studies are needed to confirm properties such as cellular uptake ability, cellular distribution, as well as to determine essential pharmacokinetic data.

## 6. Conclusions

As this review demonstrates, even though osmacycles chemistry is still in a growing stage, cyclometalated osmium compounds are essential in many aspects. The broadest chemistry of osmium and the low reactivity of osmium compounds grants determination and studies of a variety of mechanisms, which in turn provide tools to design novel complexes with a wide variety of relevant properties and applications. Due to the enormous number of molecules, known or readily accessible, that can lead to cyclometalation reactions, unique applications of osmacycles will see the light in the near future. Many cyclometalated osmium derivatives have shown remarkable catalytic activity in various reactions, principally hydrogenations and dehydrogenations, often similar and in some cases superior to those reported for analogous ruthenium systems. Such results exhibit the potential of osmacycles for the design of new highly effective and robust catalytic systems for chiral and non-chiral processes. On the other hand, new osmium-containing cyclometalated materials will continue to be studied for their activity in biological systems, and they will likely be involved in the development of innovative chemical sensors, biosensors and improved drugs.

## Figures and Tables

**Table 1:** First osmacycles prepared between 1973 and 1997.

Precursor	Cyclometalated Ligand	Osmacycle	Ref.
[Os_3_(CO)_12_]	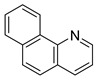 **L1**	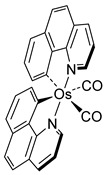 **1** speculative mixture of *cis* or *trans*	[28]
[OsH(O_2_CCF_3_)(CO)(PPh_3_)_2_]	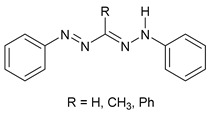 **L2**	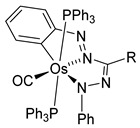 **2**	[29]
*cis*-[Os(PMe_3_)_4_H(CH_2_C(Me)_3_)]	PMe_3_ **L3**	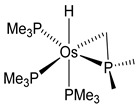 **3**	[30]
[Os(*p*-cymene)Me(Cl)(Me_2_SO)] prepared from [Os(*p*-cymene)Cl_2_]_2_	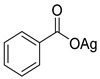 **L4**	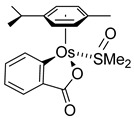 **4**	[31]
[Os(tterpy)(O)_2_(OH)(NO_3_)] prepared from K_2_[Os(O)_2_(OH)_4_]	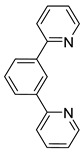 **L5**	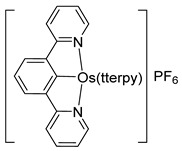 **5**	[32]
[Os(tterpy)(O)_2_(OH)(NO_3_)] prepared from K_2_[Os(O)_2_(OH)_4_]	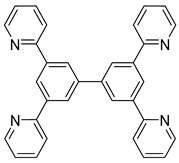 **L6**	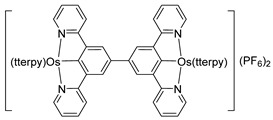 **6**	[32]
[Os(tterpy)C1_3_] prepared from [OsCl_3_]	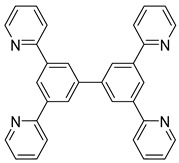 **L6**	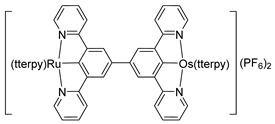 7 *	[33]
[Os(Cp)(PPh_3_)_2_]OTf	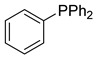 **L7**	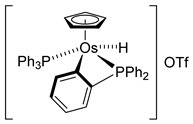 **8**	[34]
[Os(N**~**N**~**N)Cl_3_] prepared from [OsCl_3_] 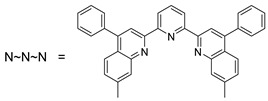	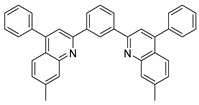 **L8**	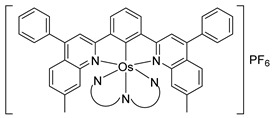 **9**	[35]

* tterpy = 4′-(4-tolyl)-2,2′:6′,2″-terpyridine.

**Table 2:** Osmacycles obtained from [OsH_6_(P*^i^*Pr_3_)_2_].

Cyclometalated Ligand			Osmacycle	Yield	Ref.
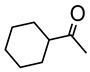 **L9**			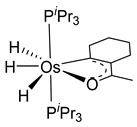 **60**	62%	[55]
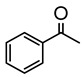 **L10**			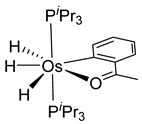 **61**	61%	[54]
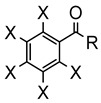 X = H, F; R = Ph, CH_3_ **L11**			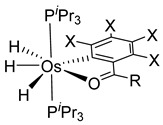 **62**	62% (X = H, R = Ph) 74% (X = F, R = CH_3_) 60% (X = F, R = Ph)	[54]
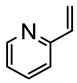 **L12**			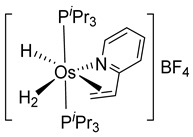 **63**	92%	[56]
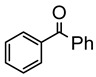 **L13**			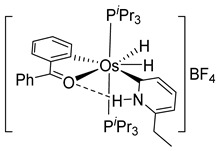 **64**	55%	[56]
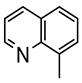 **L14**			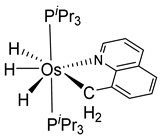 **65**	97%	[57]
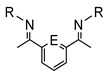 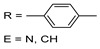 **L15, L16**			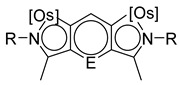 **66**	23% (E = N) 45% (E = CH)	[58]
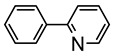 **L17**			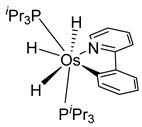 **67**	77%	[59]
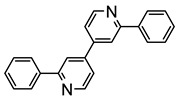 **L18**			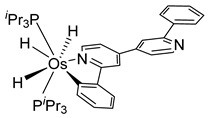 **68**	50%	[59]
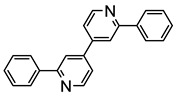 **L18**			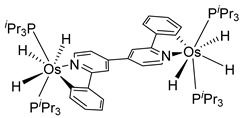 **69**	91%	[59]
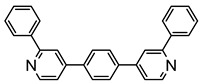 **L19**			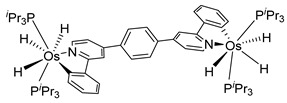 **70**	99%	[59]
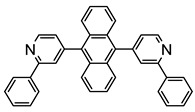 **L20**			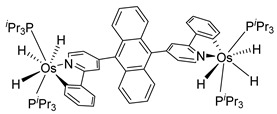 **71**	90%	[59]
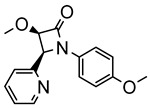 **L21**			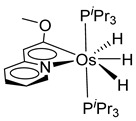 **72**	60%	[60]
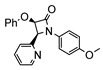 **L22**	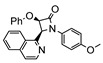 **L23**	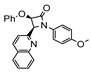 **L24**	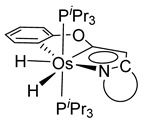 **73**	71% (**L22**) 60% (**L23**) 63% (**L24**)	[61]
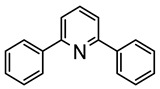 **L25**			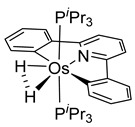 **74**	64%	[62]
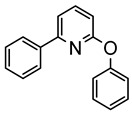 **L26**			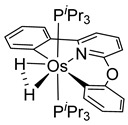 **75**	80%	[62]
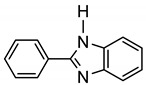 **L27**			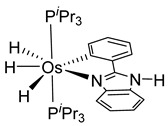 **76**	53%	[63]
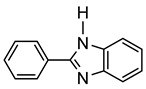 **L27**			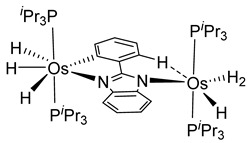 **77**	60%	[63]
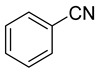 **L28**			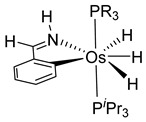 **78**	73% (R = *^i^*Pr_3_) 74% (R = Ph) R = Ph prepared from [OsH_6_(P*^i^*Pr_3_)(PPh_3_)]	[64]
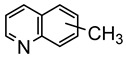 **L29**			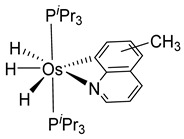 **79**	60–75%	[65]
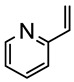 **L12**			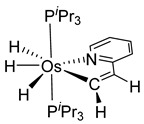 **80**	83%	[56]

**Table 3:** Reduction potential (Os^II^/Os^III^) and rate constants *k*_2_ for the electron transfer between osmium complexes and the active site of glucose oxidase (GO). Reference electrode Ag/AgCl. [Os] 20 μM, GO 1.0 × 10^−6^ M, in phosphates buffer pH 7.0. Scan rate10 mV/s [76].

Complex	*E°* (mV)	*k*_2_ (M^−1^s^−1^)
**162**	−51	0.67 × 10^6^
**163**	13	4.80 × 10^6^
**161**	32	2.00 × 10^6^
**94**	84	2.90 × 10^6^
**95**	31	1.80 × 10^6^
**159**	109	2.90 × 10^6^

## Data Availability

Not applicable.

## Abbreviation

2-MeTHF	2-methyl-tetrahydrofuran
A2780	Human ovarian cancer cell line
A549	Human lung cancer cell line
AND/OR	Boolean operator for browsing in databases
ATRP	Atom Transfer Radical Polymerization
B3LYP	Becke three-parameter exchange and Lee–Yang–Parr correlation
BJAB	Burkitt-like lymphoma
Bn	Benzyl substituent
bpy	2,2′-bipyridine
C~C’~N	Pincer ligand with Carbon-Carbon-Nitrogen, as donor atoms
C~C~C	Pincer ligand with Carbon-Carbon-Carbon, as donor atoms
C~C~C~C	Tetradentate Ligand with Carbon-Carbon-Carbon-Carbon as donor atoms
C~E	Bidentate ligand with Carbon and E as donor atoms
C~N	Bidentate ligand with Carbon and Nitrogen as donor atoms
C~N~C	Pincer ligand with Carbon-Nitrogen-Carbon as donor atoms
C~N~N	Pincer ligand with Carbon-Nitrogen-Nitrogen as donor atoms
C~N~O	Pincer ligand with Carbon-Nitrogen-Oxygen as donor atoms
C~O	Bidentate ligand with Carbon and Oxygen as donor atoms
CC_50_	50% Cytotoxic concentration
CHI/PA-1	Human ovarian carcinoma cells
cod	1,4-cyclo-octadiene
DFT	Density Funtional Theory
DMSO	Dimethyl sulphoxide
DNA	Deoxyribonucleic acid
dpbH	1,3-di(2-pyridyl)benzene
dppb	1,4-bis(diphenylphosphino)butane
E	Donor atom such as: N, P, O, S, As, Se
EC_50_	50% Effective concentration
GO	Glucose oxidase
HBMePI	1,3-bis(6′-methylpyridyl-2′-imino)isoindoline
HEK293	Kidney adenocarnocinoma cell line
HeLa	Cervical cancer cell line
HOMO	Highest Occupied Molecular Orbital
HRP	Horseradish peroxidase
IC_50_	50% Inhibitory concentration
ILCT	Intraligand Charge-Transfer
log(P_o/w_)	*N*-Octanol-water partition coefficient
LUMO	Lowest Unoccupied Molecular Orbital
Mebib	2-deprotonated form of 1,3-bis(*N*-methylbenzimidazolyl)benzene
Mebip	bis(*N*-methylbenzimidazolyl)pyridine)
MLCT	Metal-to-Ligand Charge Transfer
MT4	Leukemia cell line
N~C~N	Pincer ligand with Nitrogen-Carbon-Nitrogen as donor atoms
N~N	Bidentate ligand with Nitrogen-Nitrogen as donor atoms
N~N~N	Terdentate ligand where the atom donors are Nitrogen
Nalm6	Leukemia cell line
NHC	N-Heterocyclic Carbene
NHE	Normal Hydrogen Electrode
NMR	Nuclear Magnetic Resonance
OLED	Organic Light-Emitting Diode
Otf	Triflate
P~C(H)~P	Ligand with only phosphorous as donor atoms
P~C~P	Pincer ligand with phosphorous–carbon–phosphorous as donor atoms
PDT	Photodynamic Therapy
PhMeBIm	1-Phenyl-3-methyl-1H-benzimidazolium
phen	1,10-Phenanthroline
phpyH	2-Phenylpyridine
PMMA	Polymethylmethacrylate
ROS	Reactive Oxygen Species
SCE	Saturated Calomel Electrode
SW480	Colon adenocarcinoma cell line
TAML	Tetra-Amido Macrocyclic Ligand
TD-DFT	Time-Dependent Density Functional Theory
TOF	Turnover Frequency
tterpy	4′-tolyl-2,2′,6′,2”-terpyridine ligand
